# Graph Neural Networks for Medical Imaging Analysis and Biological Data: Integrating Topology, Geometry, Radiomics, and Generative AI

**DOI:** 10.3390/bioengineering13060638

**Published:** 2026-05-29

**Authors:** Yashbir Singh, Yassine Himeur, Colleen M. Farrelly, Peguy Kem-Meka Tiotsop Kadzue, Jennifer Z. Rozenblit, Amina Kunovac, Isabelle C. Pappas, Ashok Choudhary, Sara Salehi, Shadi Atalla, Quincy A. Hathaway

**Affiliations:** 1 Department of Radiology, Mayo Clinic, Rochester, MN 55905, USA; singh.yashbir@mayo.edu (Y.S.); 2 College of Engineering and Information Technology, University of Dubai, Academic City, Dubai 14143, United Arab Emirates; satalla@ud.ac.ae (S.A.); 3 Staticlysm LLC, Miami, FL 33157, USA; 4 Department of Mathematics, Statistics and Computer Science, Faculty of Science, The University of Bertoua, Bertoua 00237, Cameroon; kem.peguy@aims-cameroon.org; 5 School of Computer Science and Applied Mathematics, The University of the Witwatersrand, Johannesburg 2050, South Africa; 6 African Institute for Mathematical Sciences, Research and Innovation Centre, Kigali P.O. Box 6428, Rwanda; 7 Department of Mathematics, The University of Texas, Austin, TX 78712, USA; 8 UPMC Genome Center, Pittsburgh, PA 15213, USA; 9 Cooper Medical School, Rowan University, Camden, NJ 08103, USA; 10 Department of Surgery, Mayo Clinic, Rochester, MN 55905, USA; 11 Department of Radiology, University of Pennsylvania, Philadelphia, PA 19104, USA

**Keywords:** graph neural networks, medical imaging analysis, biological data, radiomics, topological data analysis, geometric deep learning, generative AI, graph transformers

## Abstract

Graph neural networks (GNNs) are increasingly used for medical imaging analysis and biological data modeling, where the integration of radiomics, topology, geometry, and generative artificial intelligence (AI) may improve representation learning from medical images and related biomedical data. Across the reviewed literature, GNNs show particular value for modeling spatial relationships, multimodal interactions, graph-structured biological networks, and non-Euclidean imaging features that are difficult to capture using conventional convolutional architectures alone. Topology- and geometry-aware approaches further expand this capability by encoding multi-scale structure, higher-order relationships, curvature, geodesic organization, and equivariant spatial priors. Hybrid graph–transformer models and generative graph methods represent emerging directions for modeling long-range dependencies, augmenting scarce datasets, supporting synthetic pretraining, and improving representation learning in low-label or heterogeneous biomedical settings. However, clinical translation remains limited by variability in graph construction, limited external validation, computational cost, scalability constraints, interpretability challenges, and uncertainty regarding the biological realism of synthetic data. Overall, this review highlights that GNN-based medical imaging analysis is most likely to advance when graph construction is biologically justified, model performance is evaluated across diverse clinical cohorts, and technical gains are paired with transparent validation, interpretability, and implementation strategies.

## 1. Introduction

Deep learning (DL) has transformed medical imaging analysis by improving image classification, segmentation, reconstruction, detection, and outcome prediction. However, many medical imaging problems are not purely grid-based. Medical images contain structured relationships among pixels, voxels, lesions, vessels, organs, cells, radiomic features, and patient-level variables. These relationships are often non-Euclidean, hierarchical, and multimodal, making medical imaging a natural domain for graph neural networks (GNNs), which model entities as nodes and their anatomical, spatial, functional, molecular, or clinical relationships as edges [1,2,3,4,5]. Therefore, this review focuses on medical imaging analysis because graph-based learning can connect image-derived features with biological structure, multimodal data, and clinically relevant prediction tasks.

The integration of topology, geometry, radiomics, and generative artificial intelligence (AI) is motivated by their complementary roles in biomedical representation learning. Radiomics provides quantitative image-derived features that can define node attributes, edge attributes, or graph-level descriptors [6]. Topology captures multi-scale connectivity, loops, voids, and higher-order relationships that may reflect tumor heterogeneity, tissue architecture, vascular branching, or brain-network organization [7,8,9,10,11,12,13,14,15,16,17,18,19,20,21,22]. Geometry encodes shape, curvature, geodesic distance, equivariant spatial priors, and symmetry constraints relevant to anatomical surfaces, pathology images, molecular structures, and orientation-dependent imaging workflows [23,24,25,26,27,28,29,30,31,32,33,34,35]. Generative AI methods, including graph autoencoders, adversarial graph generators, and graph diffusion models, can support synthetic data augmentation, graph generation, missing-edge imputation, low-label representation learning, and robustness testing [36,37,38,39,40,41,42,43]. In addition, large language models (LLMs) are increasingly relevant to biomedical imaging AI because they can support report-context integration, clinical knowledge extraction, multimodal reasoning, radiology report structuring, literature synthesis, and the construction of text-informed knowledge graphs [44,45,46]. Thus, this review considers GNNs as a framework for integrating image-derived, biological, structural, geometric, generative, and language-derived information.

**Figure 1:**
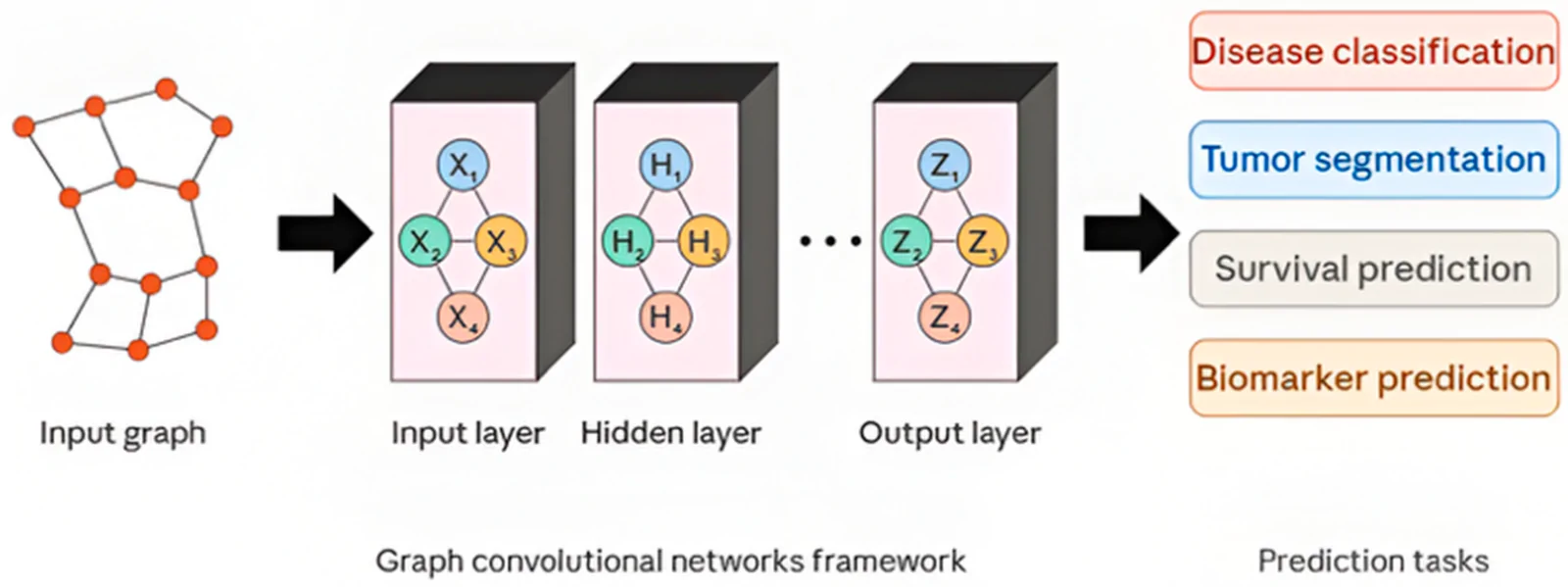
Conceptual baseline for GNN-based medical image analysis.

GNNs are DL architectures designed to process data represented as graphs [4,5,47,48,49]. A typical GNN converts biomedical data into a network structure, processes node and edge features through graph convolution, message passing, attention, or related operations, and produces outputs for prediction, classification, or representation learning. Because later sections discuss multiple specialized GNN variants, Figure 1 is intended as a simplified conceptual baseline that introduces the common graph-learning workflow before discussing spectral, spatial, attention-based, topology-aware, geometry-aware, generative, and hybrid graph–transformer extensions.

Medical images or related biomedical data are represented as graphs in which nodes may correspond to pixels, voxels, regions of interest, anatomical structures, cells, radiomic features, molecular entities, or patient-level variables, and edges encode spatial, anatomical, functional, molecular, or clinical relationships. This figure introduces the general GNN workflow, graph construction, node/edge feature learning, message passing, and prediction, before later sections discuss specialized GNN variants and hybrid architectures.

GNNs support node-, edge-, and graph-level prediction tasks, including node classification, link prediction, edge classification, graph classification, similarity analysis, and community detection [50,51,52,53,54,55,56,57,58]. In biomedical imaging, these tasks can correspond to classifying anatomical regions or cells, predicting relationships among structures or biomarkers, assigning diagnoses or outcomes to patient-level graphs, or identifying disease-associated subgraphs. These capabilities motivate the use of GNNs in radiomics, multimodal imaging, molecular modeling, pathology, neuroimaging, cardiovascular modeling, and clinical outcome prediction.

This review makes five specific contributions. First, it summarizes how medical images and biological data can be transformed into graph representations through node definition, feature extraction, edge construction, and graph structuring. Second, it compares major GNN model families, including spectral, spatial message-passing, attention-based, temporal, heterogeneous, topology-aware, geometry-aware, generative, and hybrid graph–transformer approaches. Third, it explains how topology, geometry, radiomics, and generative AI contribute complementary information to biomedical graph learning, including image-derived quantitative features, multi-scale structure, higher-order relationships, spatial priors, and synthetic graph or image augmentation. Fourth, it reviews representative applications across oncology, neuroimaging, dementia, cardiovascular modeling, ECG analysis, and coronary artery analysis [59,60,61,62,63,64,65,66,67,68,69,70,71,72,73,74,75,76,77,78,79,80,81,82,83,84,85,86,87,88,89,90,91,92,93,94,95,96,97,98,99,100,101,102,103,104,105]. Fifth, it synthesizes implementation considerations relevant to clinical translation, including data modality, computational cost, scalability, interpretability, clinical suitability, graph construction variability, and validation limitations. These contributions position the review as a clinically oriented synthesis of contemporary GNN methods for medical imaging analysis and biological data modeling rather than as a general survey of GNN architectures.

## 2. Background of GNNs

**Figure 2:**
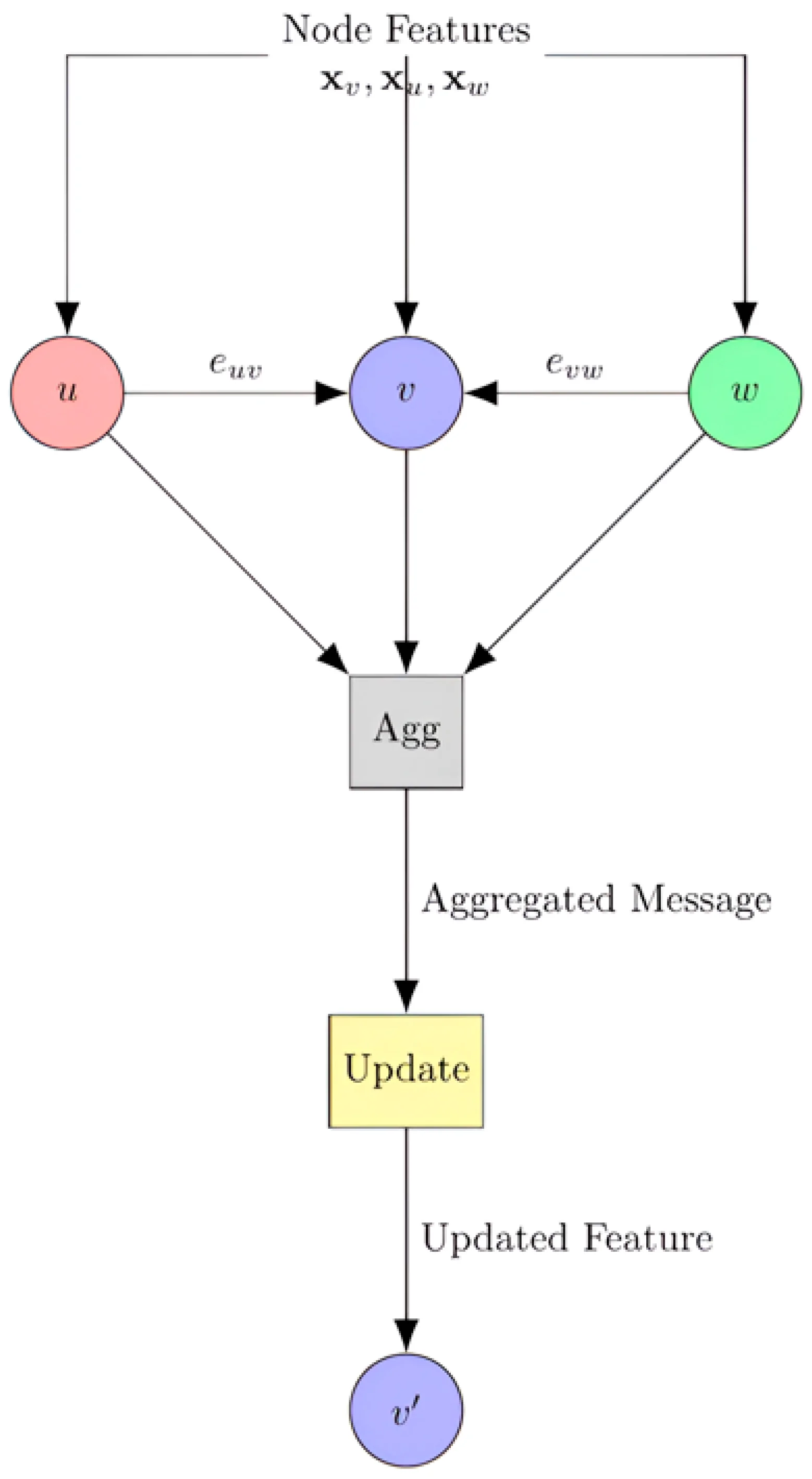
Illustration of the GNN message passing mechanism.

GNNs are designed to learn from graph-structured data, where a graph G=(V,E) consists of nodes, edges, and associated node or edge features; where a graph *G* = (*V*, *E*) consists of a set of vertices V, a set of edges E, and a feature vector xv for each vertex v ∈ V [47,48,49] (Figure 2). In biomedical imaging and biological data analysis, nodes may represent pixels, voxels, regions of interest, anatomical structures, cells, molecular entities, radiomic features, or patient-level variables, while edges may encode spatial proximity, anatomical adjacency, functional connectivity, molecular interaction, feature similarity, or clinical relationships. Most GNNs update node representations through message passing, in which each node aggregates information from neighboring nodes using a permutation-invariant function and then applies a learnable update rule [49,106,107,108,109]. These operations can be trained in supervised, semi-supervised, or unsupervised settings depending on label availability [110]. Common variants, including graph convolutional, attention-based, autoencoding, and temporal architectures, adapt this general framework to different graph structures, data modalities, and prediction tasks [111]. This brief background is included only to define the terminology needed for the taxonomy, image-to-graph transformation, topology-aware, geometry-aware, generative, and clinical application sections that follow.

### Literature Search Strategy

This article was designed as a structured narrative review rather than a formal systematic review or meta-analysis. To improve transparency, relevant articles were identified through targeted searches of PubMed/MEDLINE, IEEE Xplore, Scopus, Web of Science, Google Scholar, and arXiv using combinations of terms related to graph-based learning, biomedical imaging, topology, geometry, radiomics, and generative AI. Search terms included “graph neural network”, “graph convolutional network”, “graph attention network”, “message passing neural network”, “geometric deep learning”, “medical imaging”, “radiology”, “radiomics”, “neuroimaging”, “cardiovascular imaging”, “topological data analysis”, “persistent homology”, “generative graph model”, and “graph diffusion model”. Searches prioritized peer-reviewed articles, major conference papers, and highly relevant preprints published through 2025, with additional targeted searches to incorporate recent hybrid graph–transformer, generative graph, topology-aware, and geometry-aware GNN methods. Articles were included when they introduced, reviewed, or applied graph-based methods relevant to medical imaging, radiomics, multimodal biomedical data, or biomedical representation learning. Articles were excluded when they were not biomedical or imaging-relevant, did not involve graph-based learning, lacked sufficient methodological detail, or duplicated content from more recent or more comprehensive sources.

## 3. Taxonomy of Existing GNN Models

GNNs have evolved into a powerful tool for handling graph-structured data, leading to the development of diverse models based on the type of graph convolution employed. These models can broadly be categorized into spectral and spatial approaches, each leveraging different methodologies to process graph data [112]. In spectral graph learning, a graph G=(V,E) with adjacency matrix A and degree matrix D can be represented by the graph Laplacian L=D−A , or by the normalized Laplacian $L_{\mathrm{norm}}=I-D^{-1/2}AD^{-1/2}$ . The eigendecomposition $L=U\Lambda U^{⊤}$ defines a graph Fourier basis, allowing graph convolution to be interpreted as filtering node features over graph frequencies. Table S1 summarizes the major GNN model families, graph-learning strategies, biomedical relevance, and representative references discussed in this section [50,51,52,53,54,55,56,57,58,113,114,115,116,117,118,119,120,121,122,123,124,125,126,127,128,129,130,131].

Hybrid graph–transformer architectures represent a more recent extension of attention-based GNNs. These models combine the local relational inductive bias of GNNs with the global context modeling of transformer-based self-attention. In biomedical imaging, this design may be useful when local anatomical structure and long-range biological dependencies are both important, such as in brain-region graphs, white-matter tractography, pathology cell graphs, vascular structures, radiomics-derived region graphs, and multimodal patient-level graphs [127,130,131,132,133,134,135,136].

## 4. Training Techniques

Training strategy and scalability strongly influence whether GNNs can be applied to biomedical imaging and biological data. Because labels are often limited, heterogeneous, or expensive to obtain, biomedical GNNs may use supervised, semi-supervised, unsupervised, or self-supervised learning depending on the task and label availability. Scalability is also important because imaging-derived graphs, molecular networks, pathology cell graphs, and multimodal patient graphs may contain large numbers of nodes, edges, or modalities. Table S2 summarizes the major training paradigms, scalability strategies, and advanced GNN extensions discussed in this section [137,138,139,140,141,142,143,144,145].

## 5. Data Transformations: Images to Graphs

The transformation of images into graphs facilitates the representation of complex spatial relationships and features within images in a graph structure, capitalizing on the inherent advantages of GNNs in capturing and analyzing data relationships [146]. The process encompasses several key steps:

### 5.1. Node Identification

Initially, the process requires the identification of nodes within the image. Depending on the application, nodes can represent pixels, regions of interest (ROIs), or semantic features derived from the image. In the context of medical imaging, nodes may denote distinct anatomical structures (e.g., heart, lungs, trachea, esophagus) or areas indicative of pathological changes (e.g., nodules, tumors, calcified vasculature, implanted hardware) [147].

### 5.2. Feature Extraction

Each node is associated with a set of features, ranging from simple pixel intensity values to complex high-level descriptors obtained using traditional image processing techniques or DL models. The selection of features depends on the specific task and the characteristics of the image data [147]. Texture-based features derived through radiomics may also be utilized.

### 5.3. Edge Construction

Edges are established between nodes to depict relationships or interactions among them, such as spatial proximity or feature similarity. The basis for edge construction can vary extensively, including spatial proximity, similarity in feature space, or predefined connections informed by domain knowledge. For example, in social network images, edges could link individuals who are physically proximate [147]. In medical imaging, edges could represent proximally adjacent anatomical structures or structures that share morphological (e.g., a pair of kidneys) or textural (e.g., spleen and liver) similarities.

### 5.4. Graph Structuring

**Figure 3:**
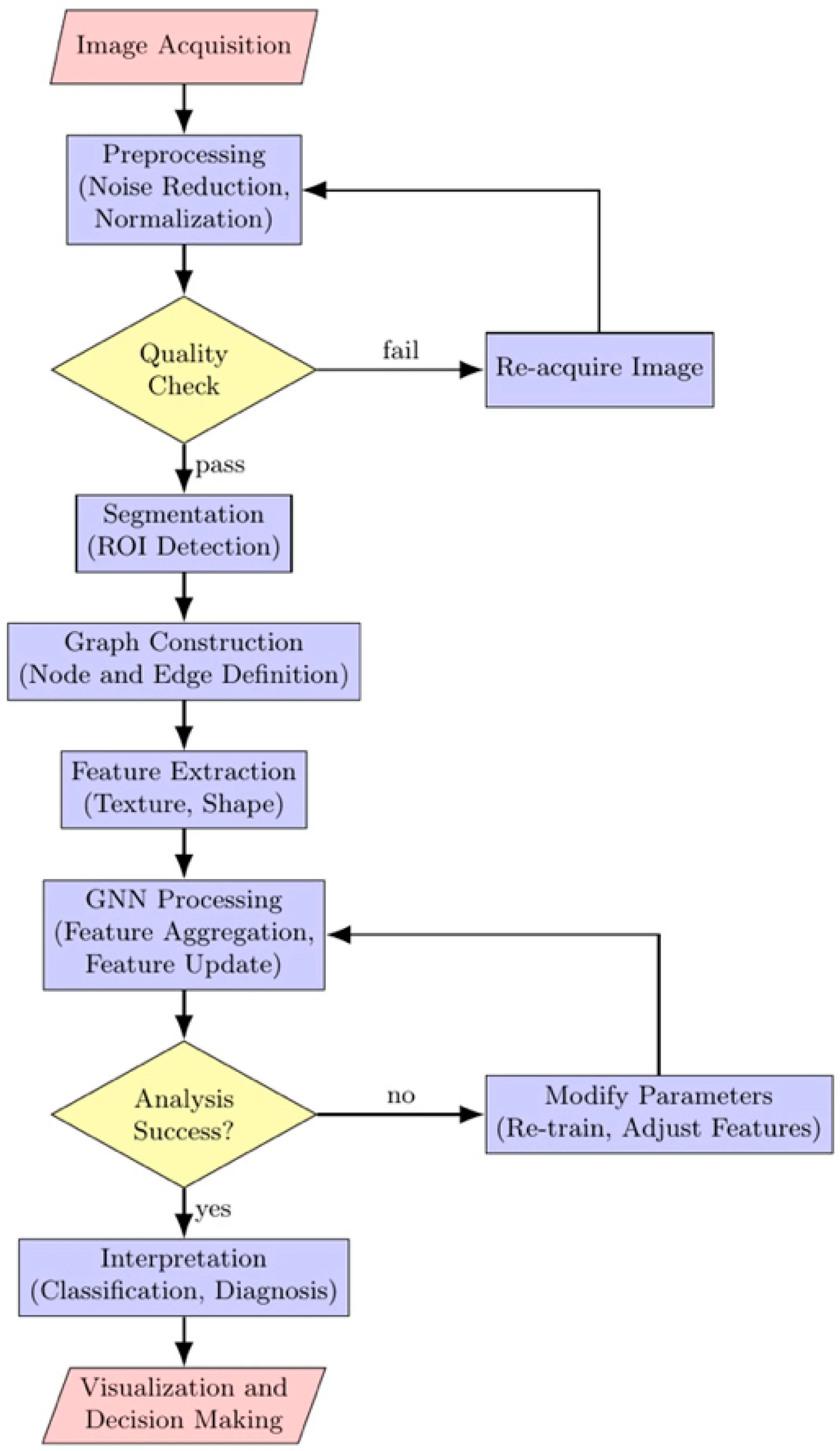
Detailed flowchart of the data transformation process from images to graphs using GNNs in medical image analysis.

Once the nodes and edges are defined, the image is effectively transformed into a graph structure. This graph may be directed or undirected, weighted or unweighted, depending on the nature of the relationships it portrays. Graph-level features may also be delineated to reflect the global properties of the image [147]. Figure 3 presents a detailed flowchart of the data transformation process from images to graphs using GNNs in medical image analysis.

## 6. Generative AI for Graph-Based Biomedical Imaging Analysis

Generative AI can complement GNNs by augmenting scarce biomedical data, generating plausible graph structures, and supporting self-supervised or latent-space representation learning. In graph-based medical imaging workflows, generative models may operate upstream on images or segmentations, on extracted radiomics features, or directly on graph representations. Graph-specific generative models include variational graph autoencoders (VGAEs), which use graph-convolutional latent-variable learning [132]; GraphVAE, which decodes probabilistic graph structures [133]; GraphRNN, which generates graphs through autoregressive node–edge formation [134]; MolGAN, which adapts adversarial learning to molecular graph generation [135]; and graph diffusion models, which use denoising-based graph synthesis for discrete node and edge attributes [37,136]. Broader biomedical graph-learning frameworks have also emphasized the relevance of generative graph models for representation learning, data augmentation, and biomedical discovery [38].

These methods are relevant to biomedical imaging because cell-neighborhood graphs, brain-connectivity graphs, vascular graphs, molecular interaction graphs, and radiomics-derived region-adjacency graphs are often small, imbalanced, privacy-constrained, or difficult to annotate. Generative graph models may therefore support graph augmentation, synthetic pretraining, missing-edge imputation, robustness testing, or class balancing before downstream GNN training [37,38,132,133,134,135,136]. However, synthetic data should be validated for both structural realism and clinical utility, including node-feature distributions, edge distributions, graph topology, calibration, subgroup performance, external validation, and interpretability. Synthetic-to-real domain shift, hallucinated anatomy, biologically implausible graph topology, and bias amplification remain important limitations.

### Broader Biomedical Graph Applications: Epidemic and Infodemic Networks

Although this review focuses primarily on medical imaging analysis and biological data modeling, recent biomedical GNN applications also extend to population-level public-health and information-network settings. DeepTrace models digital contact tracing as online graph exploration and uses a GNN to learn maximum-likelihood source estimation from iteratively sampled epidemic networks, supporting forward/backward tracing and superspreader identification in COVID-19 contact networks [148]. MEGA similarly frames infodemic risk management as a graph analytics problem, combining machine-learning-enhanced graph features with GNN-based modeling to support risk management in biomedical information networks [149]. These studies broaden the biomedical scope of GNNs by demonstrating their relevance not only to image-derived anatomical and radiomic graphs, but also to epidemic transmission networks, infodemic information networks, and population-scale public-health systems.

## 7. Topology-Based GNNs

Topology is a branch of mathematics concerned with the global properties of spaces and objects, such as the number of holes or the number of unique paths defined on the space or object. Topological properties can be defined on discrete spaces, such as graphs, to understand important structural features of those spaces. Within the context of GNNs, computing topological properties of the graph being fed into the neural network—or properties of the neural network architecture—allows one to quantify properties important for graph embedding or pooling layers, which can improve algorithm performance.

### 7.1. Persistent Homology GNNs

**Figure 4:**
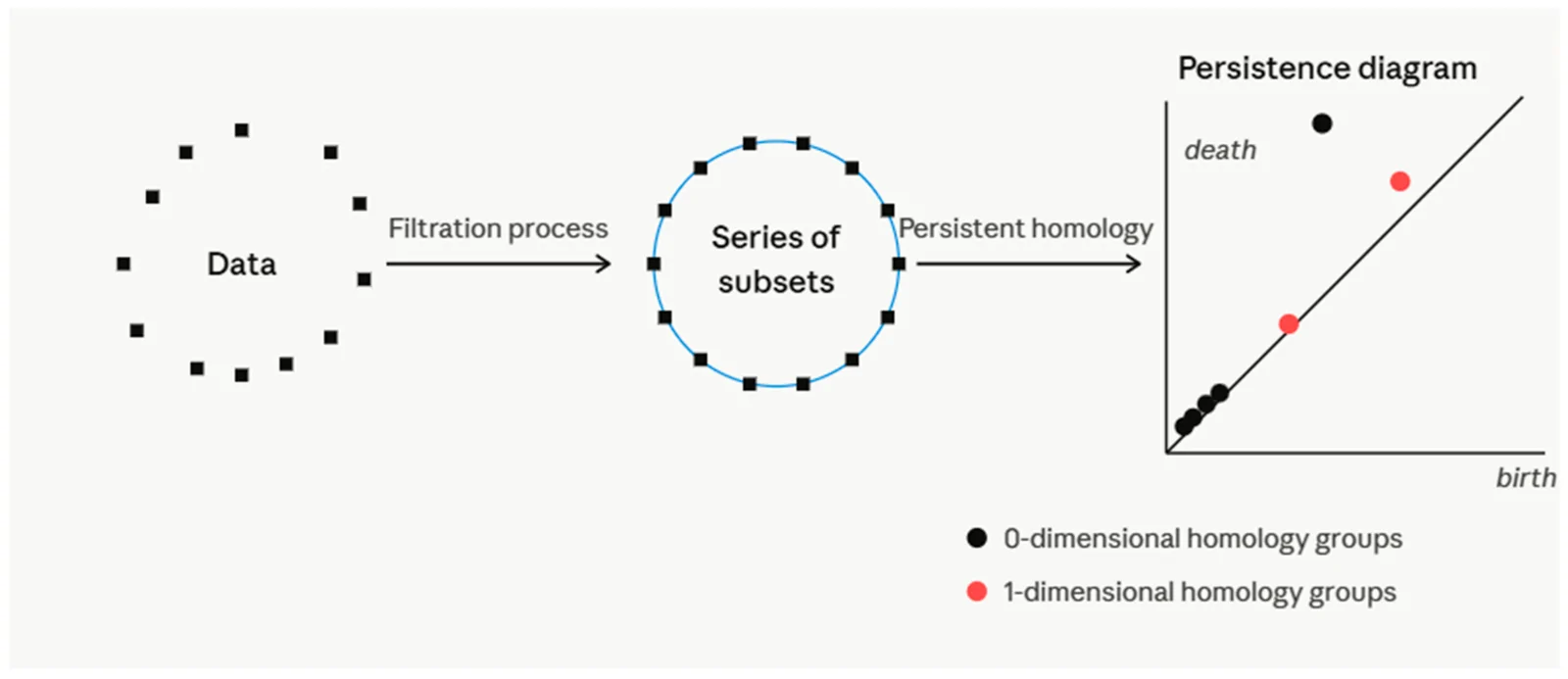
Overview of persistent homology where data points are connected based on distance filtration and topological features are tracked across the full filtration.

Persistent homology (PH) is a primary tool in topological data analysis (TDA) that captures topological features across different spatial resolutions. It fundamentally operates through two processes: filtration, in which data are progressively simplified into a series of subsets using a distance metric, and the tracking of topological features—such as loops or holes—across these subsets (Figure 4). PH constructs a filtration $X_{0}\subseteq X_{1}\subseteq \ldots \subseteq X_{t}$ , where each $X_{i}$ reflects the data at a different distance, similarity, or intensity threshold. Across this filtration, homology groups $H_{k}(X_{i})$ track k -dimensional topological features, including connected components $\left(H_{0}\right)$ , loops $\left(H_{1}\right)$ , and voids $\left(H_{2}\right)$ . These features are summarized in a persistence diagram [7]. The persistence diagram tracks the “birth” and “death” of the 0th, 1st, and higher-dimensional homology groups (connected components, loops, and the higher-dimensional “holes” of the graph data studied, respectively). This methodology enables the systematic breakdown of complex data structures, such as medical imaging data, into interpretable topological summaries [8,9].

Filtration is a sequence of data objects, wherein each object represents a data snapshot at a certain threshold of granularity, revealing inner structures and connections. During filtration, data points within the thresholded distance of each other—either pairwise in the Vietoris–Rips complex or mutually in the Cech complex—are connected into data objects with vertices, edges, and their higher-dimensional analogues. This provides a sequence of nested objects whose features can be summarized and compared across distances. PH provides a rich, multi-scale representation of the data’s inherent topology by examining these data objects. For instance, when considering a set of points from medical imaging containing malignancy, PH aids in understanding how tumor cells cluster or disperse as the distance threshold changes, offering insights into the tumor’s morphological characteristics.

GNNs [10,11], particularly those based on the message-passing paradigm [11], cannot capture the global topological features of graphs, which are crucial for tasks requiring a nuanced appreciation of graph connectivity, such as in medical image analysis and computational biology [12,13]. Integrating PH into GNNs equips the network with a robust mechanism to encode not only the data’s immediate features but also its intrinsic topological properties. This integration is particularly groundbreaking in radiomics, where understanding the underlying biological structures within medical images is crucial. For example, in analyzing tumor heterogeneity, PH-GNNs [14] can discern subtle topological variations within the tumor microenvironment that may correlate with clinical outcomes. A network enriched with topological data becomes adept at identifying patterns that traditional analysis might overlook, such as the spatial arrangement of tumor cells or the interplay between different tumor regions—both crucial for accurate diagnosis and treatment planning.

The workflow from raw medical images to actionable insights using PH-GNNs involves several steps. Initially, the image data are transformed into a point cloud or graph structure, wherein each point or node represents a specific feature or region in the image. Subsequently, PH is employed to extract topological features, which are then integrated into the GNN framework. There have been significant innovations in the integration of PH within GNNs, introducing four key layers: TOGL by Horn et al., 2021 [12], TRI-GNN by Chen et al., 2021 [15], TREPH by Ye et al., 2023 [16], and TTG-NN by Wen et al., 2024 [17], each embedding topological insights into GNNs based on PH principles. However, due to the computational cost of PH, these topological layers struggle to handle higher-order networks such as simplicial complexes, which are crucial for effectively modeling complex data in fields like neuroscience and radiomics.

### 7.2. Simplicial Neural Networks

**Figure 5:**
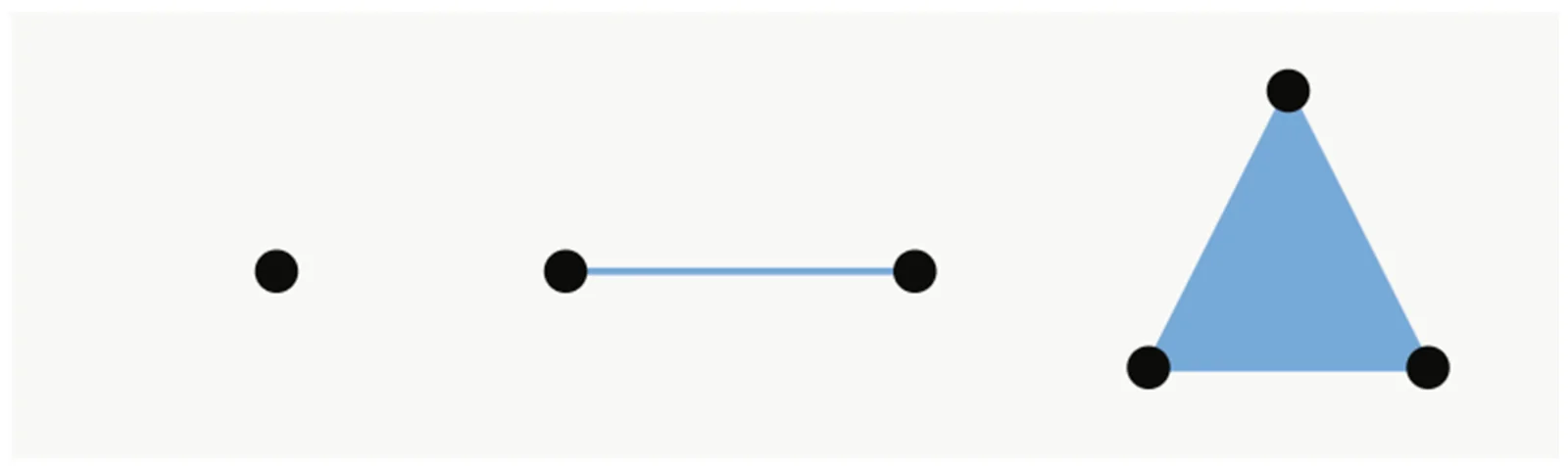
Simplices of dimension 0 (point on left) through 2 (triangle on right).

Simplicial complexes are topological spaces formed by simplices, which include points (0-simplexes), line segments (1-simplexes), triangles (2-simplexes), and their higher-dimensional counterparts (Figure 5). They extend graphs by including higher-order connections, encapsulating relationships among multiple nodes. For example, a triangle (2-simplex) in a simplicial complex can represent a mutual interaction among three nodes simultaneously. This is particularly useful in radiomics, where interactions among multiple regions of interest (ROIs) or features can influence diagnosis or treatment outcomes.

Simplicial neural networks (SNNs) [18] represent an advanced generalization of GNNs, extending their applicability to data structured on simplicial complexes. Unlike graphs, which primarily encapsulate pairwise relationships through edges, simplicial complexes allow for the encoding of higher-order interactions, capturing the multifaceted relationships inherent in complex data. This framework enables SNNs to harness higher-dimensional topological information, offering a richer and more descriptive data representation than traditional GNNs.

A recent study (Elbi et al. [18]) considered complexes created from co-authorship citation data and highlighted SNNs’ effectiveness in imputing missing citations, significantly outperforming traditional methods. By introducing data missingness at rates from 10% to 50%, SNNs demonstrated superior accuracy in data imputation tasks, particularly with 30% missing data, where their mean accuracies far exceeded those of baseline methods.

SNNs are particularly well-suited for tasks where the data’s intrinsic structure is complex and where relationships extend beyond simple pairwise interactions. However, SNNs often share PH’s computational challenges. One notable development is the Hodge-Laplacian Heterogeneous Graph Attention Network (HL-HGAT), a version of simplicial neural networks that processes simplicial network neighborhoods via the Hodge Laplacian [19]; higher-order topological learning further generalizes graphs to simplicial or cellular complexes, allowing models to represent interactions among more than two entities. In this setting, boundary operators $B_{k}$ map k -dimensional simplices to their (k−1) -dimensional faces, and the k -th Hodge Laplacian can be written as $L_{k}=B_{k}^{⊤}B_{k}+B_{k+1}B_{k+1}^{⊤}$ . Whereas the standard graph Laplacian supports signal processing over nodes, Hodge-Laplacian operators support signal processing over edges, faces, and higher-dimensional structures. This provides a means to filter and weight neighborhoods across dimensions, reduce complexity in each layer, and pool results in a way that allows visualization.

The results of HL-HGAT are promising from a medical imaging perspective [19]. HL-HGAT outperformed state-of-the-art CNNs on image classification tasks, showing greater spatial separation of classes in processing layers, better accuracy, and better computational efficiency. When tested on fMRI datasets benchmarked against GNNs specifically designed for brain imaging, HL-HGAT significantly outperformed state-of-the-art GNNs by a large margin, and the pooling operation provided a substantial speed-up and reduction in memory usage.

### 7.3. Cellular Complex Neural Networks

**Figure 6:**
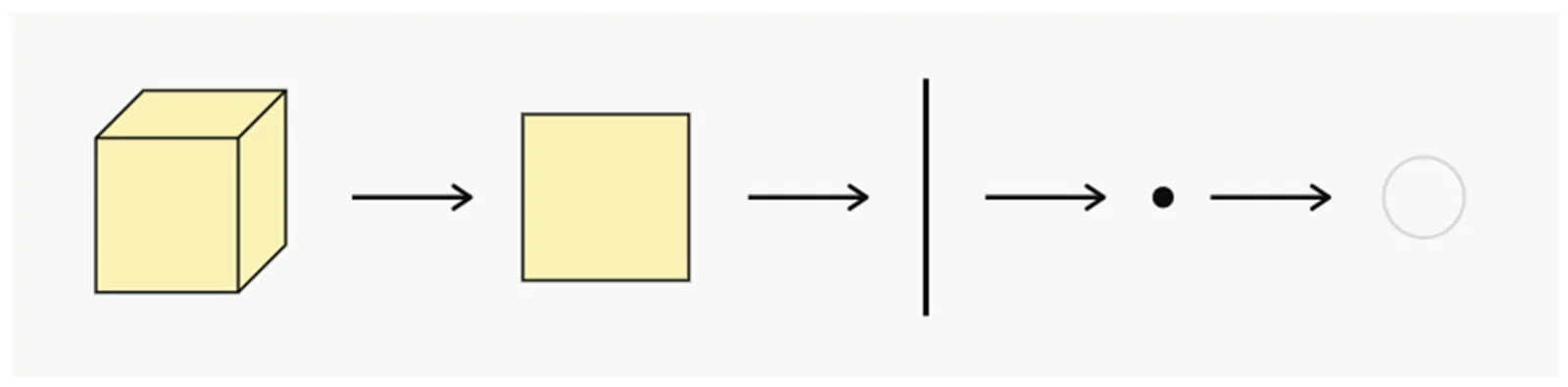
A 3-cube (topologically equivalent [homeomorphic] to a 3-dimensional ball), a 2-cube (homeomorphic to a 2-dim ball), a 1-cube (line), a 0-cube (point), and the (−1)-cube.

One extension of the simplicial neural network framework is cellular complex neural networks [20]. Cellular complexes are topological spaces of a given dimensionality that are homeomorphic to topological balls (Figure 6). Cellular complex neural networks employ a specialized message-passing scheme to aggregate and update weights related to cellular complex neighborhoods during network training.

Cellular complex neural networks can model hierarchical structures across cell dimensions, allowing them to capture more nuanced information about neighborhoods in the network than standard GNNs, thereby improving performance through an encoder–decoder embedding strategy. In addition, they demonstrate greater computational efficiency than simplicial complex neural networks [20].

### 7.4. Sheaf Neural Networks

Another topological extension of GNNs involves replacing the graph Laplacian with the sheaf Laplacian, which maps vertex–edge pair information into a new space [21]. The sheaf Laplacian allows one to understand the boundaries of the graph and flow properties on these boundaries, yielding information about the connectivity and orientation of the entire graph. Sheaf Laplacians can be computed through polynomial basis approximations, providing a computationally feasible implementation. They tend to outperform graph Laplacians when edges are signed or asymmetric, when vertex properties are non-constant across the graph, or when input feature noise levels are high [21].

**Figure 7:**
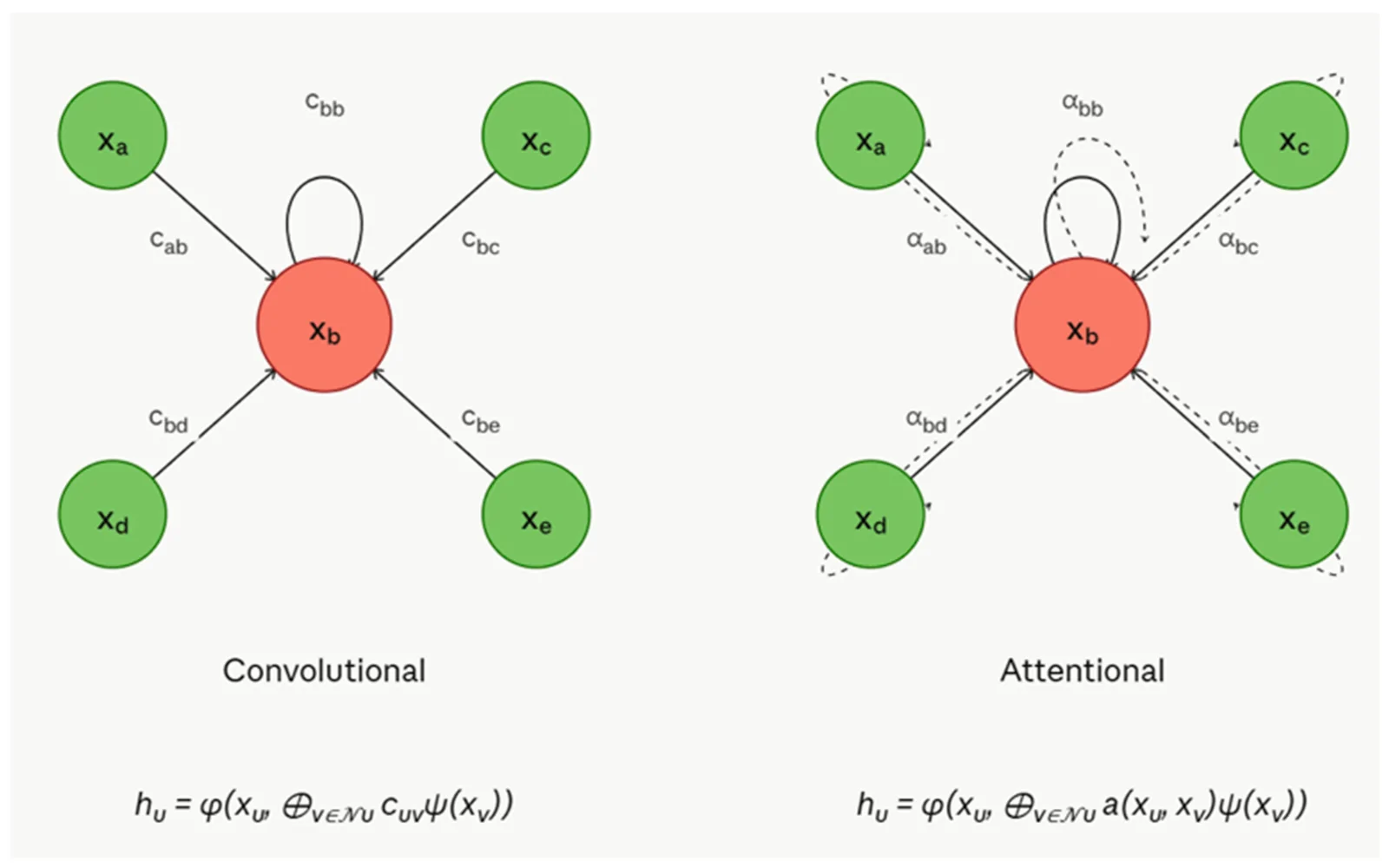
Attentional GNNs can represent convolutional GNNs by an attention mechanism implemented as a look-up table a(xu, xv) = cuv. Both convolutional and attentional GNNs are special cases of message-passing where the messages are only the sender nodes’ features.

Figure 7 illustrates the conceptual framework wherein attentional GNNs emulate the behavior of convolutional GNNs through an attention mechanism. This mechanism is represented as a look-up table, a(xu, xv) = cuv, effectively classifying both network types as subsets of message-passing architectures. Convolutional GNNs use a constant coefficient cuv to scale the features of the sender node xv, while attentional GNNs dynamically adjust this scaling through an attention coefficient a(xu, xv) that depends on both the sender and receiver nodes.

### 7.5. Torsion GNNs

Another intriguing avenue at the intersection of topology and GNNs is homotopy, a topological property that determines whether one space can be continuously deformed into another without cutting the space. Analytic torsion is a computational tool for determining whether spaces are homotopy equivalent. Within the context of GNNs, analytic torsion can be estimated as an alternating sum of the Hodge Laplacians of the graph [22] and is then used to modify the message-passing step by weighting neighborhood vertices locally based on the simplices to which each vertex belongs. Torsion GNNs are competitive with state-of-the-art algorithms on many types of vertex classification tasks and work well with low-dimensional simplicial complex computations, suggesting high efficiency in practice [75].

### 7.6. Geometry-Based GNNs

**Figure 8:**
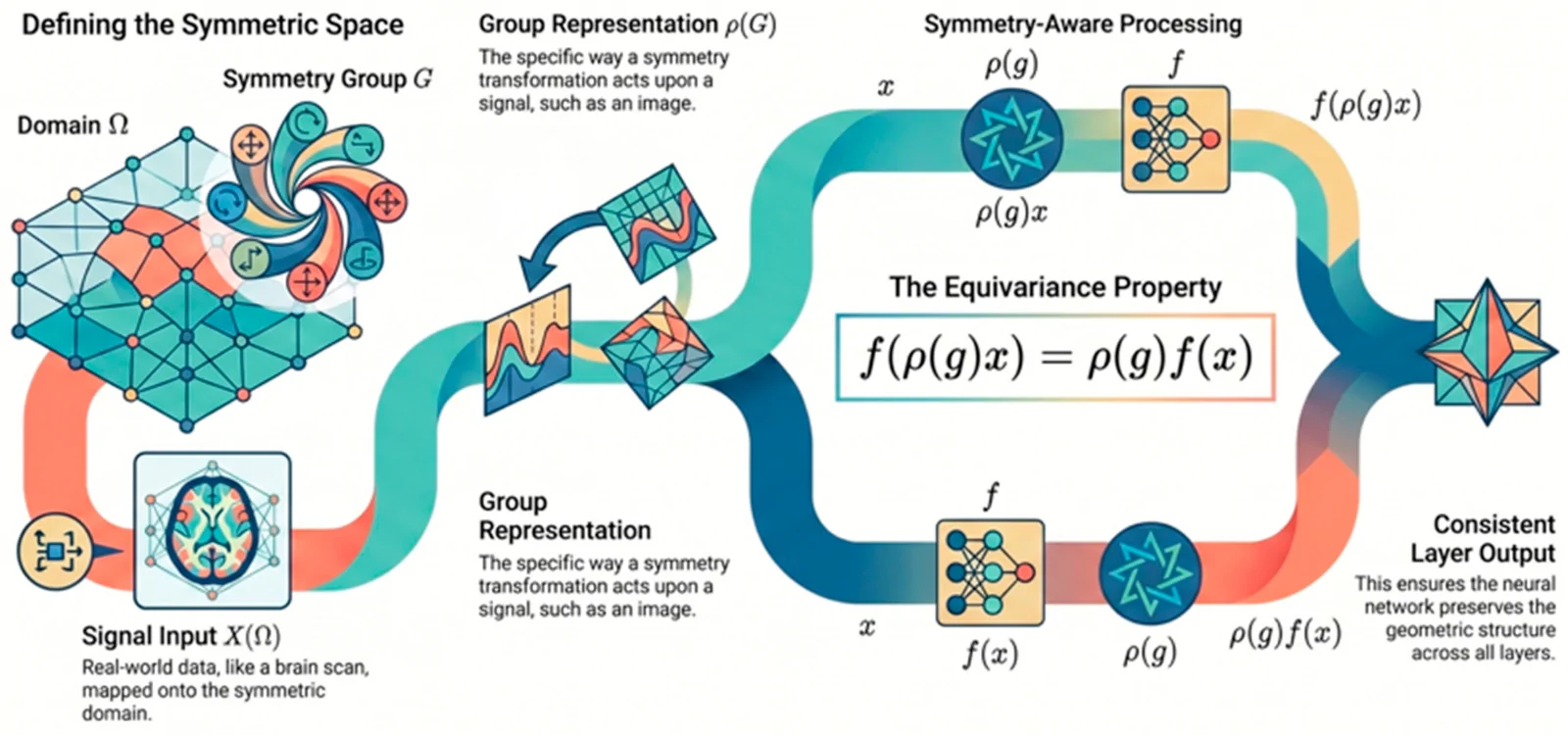
Geometric DL where symmetry is imposed across layers of the neural network.

Whereas topology refers to mathematical tools that quantify the global properties of a space or object, local properties are measured by the tools of differential geometry. Geometry-aware GNNs emphasize spatial structure, symmetry, curvature, and equivariance. A model f is equivariant to a transformation group G if f(g⋅x)=g⋅f(x) for all transformations g∈G . This property ensures that transformations of the input, such as rotation, translation, reflection, or permutation, produce corresponding transformations of the output representation. For graphs, permutation equivariance is especially important because node ordering is arbitrary; if P is a permutation matrix, a permutation-equivariant GNN satisfies $f(PAP^{⊤},PX)=Pf(A,X)$ . Because pooling depends on local neighborhoods, incorporating geometric properties may improve feature aggregation (Figure 8).

### 7.7. Curvature GNNs

One important tool of differential geometry is the measurement of curvature. Ricci curvature measures how much a space deviates from a flat object as one travels along its surface. One discrete instance, called the Ollivier–Ricci curvature, measures the expansion or contraction of a space by comparing distances [23]. Curvature can be leveraged in message-passing steps to normalize local neighborhoods by weighting vertices or edges according to curvature metrics, making GNN layers more computationally efficient [23,24]. For larger and denser graphs, curvature-aware GNNs tend to outperform other GNN variants [23].

### 7.8. Equivariant GNNs

**Figure 9:**
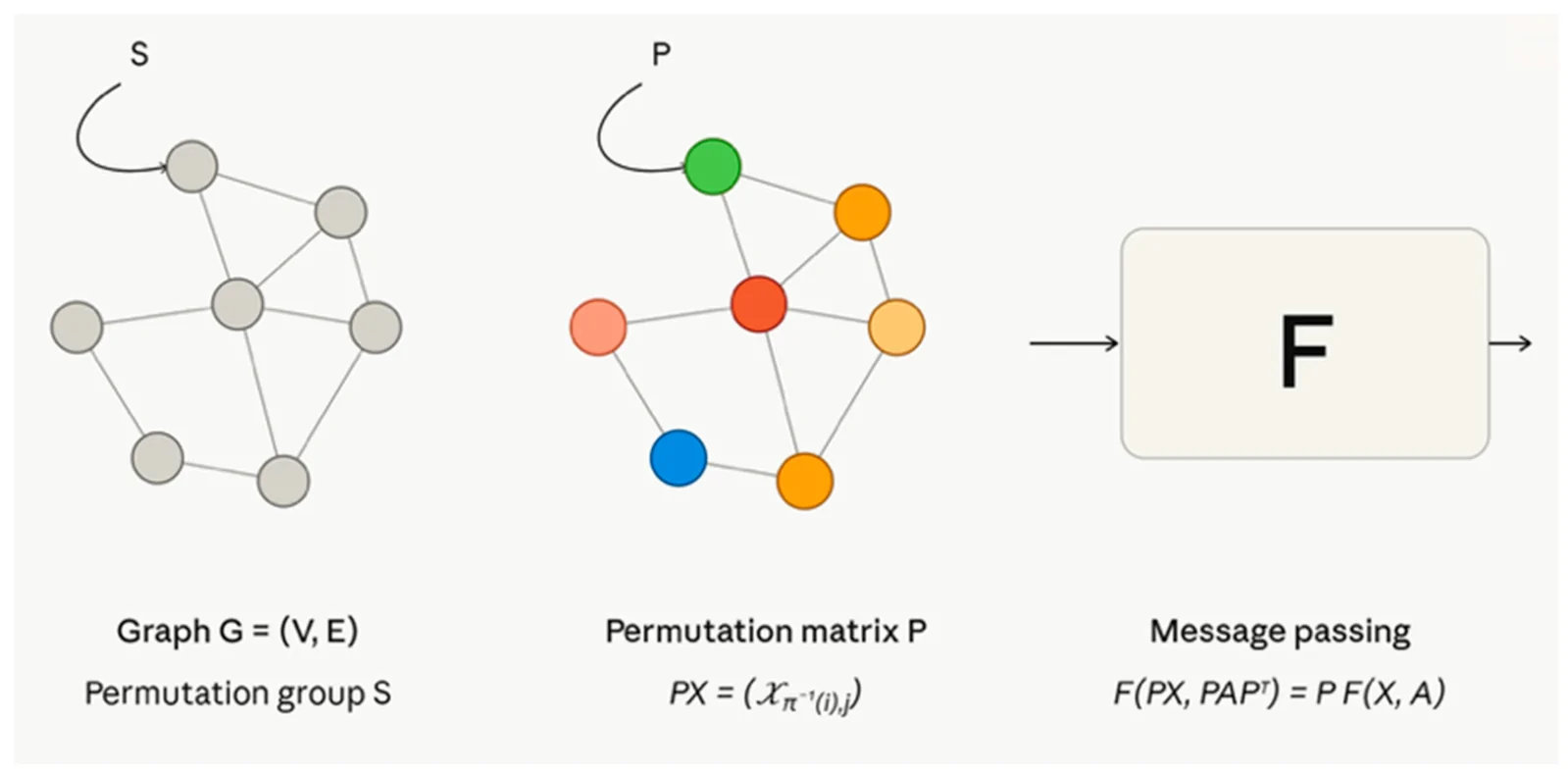
Equivariant GNNs, where neighborhood message passing does not depend on graph label ordering but rather on the graph structure.

One burgeoning field of geometric applications in neural networks involves Lie algebra, a branch of mathematics that deals with symmetries and relationships of a space or object that leave certain properties of the object intact [25,26]. Pathology slide data often include rotated or translated images due to mounting procedures and image capture conditions. Therefore, Lie-algebra-based GNNs and CNNs theoretically address potential issues in analyzing pathology slide data using neural networks [27]. Figure 9 presents equivariant graph neural networks, wherein neighborhood message passing does not depend on graph label ordering but rather on the graph structure itself.

Rotations and other Euclidean transformations (such as reflections and translations) are the most commonly encountered types of symmetries in neural network applications of Lie algebra. Lie groups must be closed under composition, possess an identity transformation, have inverses for all elements, and satisfy an associative composition law [25]. Neural networks and preprocessing steps based on Lie algebra are becoming more prevalent in signal processing [28], tensor network creation [29], toxicity prediction [30], and image data augmentation [31].

LieConv is a GNN with a predefined Lie algebra structure imposed on its convolution layer [25], creating an equivariance property in that layer. Equivariance ensures that group operations applied to layer inputs are equivalently applied to layer outputs. LieConv builds group convolution layers via a lifting process in which group convolutions are applied to raw inputs through a Monte Carlo simulation [25]. Equivariant GNNs (based on network symmetries over rotations, translations, reflections, and permutations) define group-based maps from the data space to other data spaces that preserve edge, aggregation, and node symmetries across spaces in the convolution layer [32], achieving state-of-the-art performance on molecular data benchmarks with low computational cost [32].

One major limitation of many Lie-algebra-based GNNs is the requirement to define Lie groups before constructing the GNN [26,33]. L-conv automatically discovers discrete and continuous symmetry groups in the data as part of the CNN and GNN design process [33]. LieGAN [26] builds on this concept by adding a GAN step to discern the best-fitting Lie group based on data input before constructing an equivariant GNN. When tested on a particle jet classification task, LieGAN not only recovered a highly complex Lie group as the best-fitting symmetry match, but also outperformed the Satorras et al. [32] equivariant GNN and achieved state-of-the-art results on that classification task.

### 7.9. Geodesic GNNs

**Figure 10:**
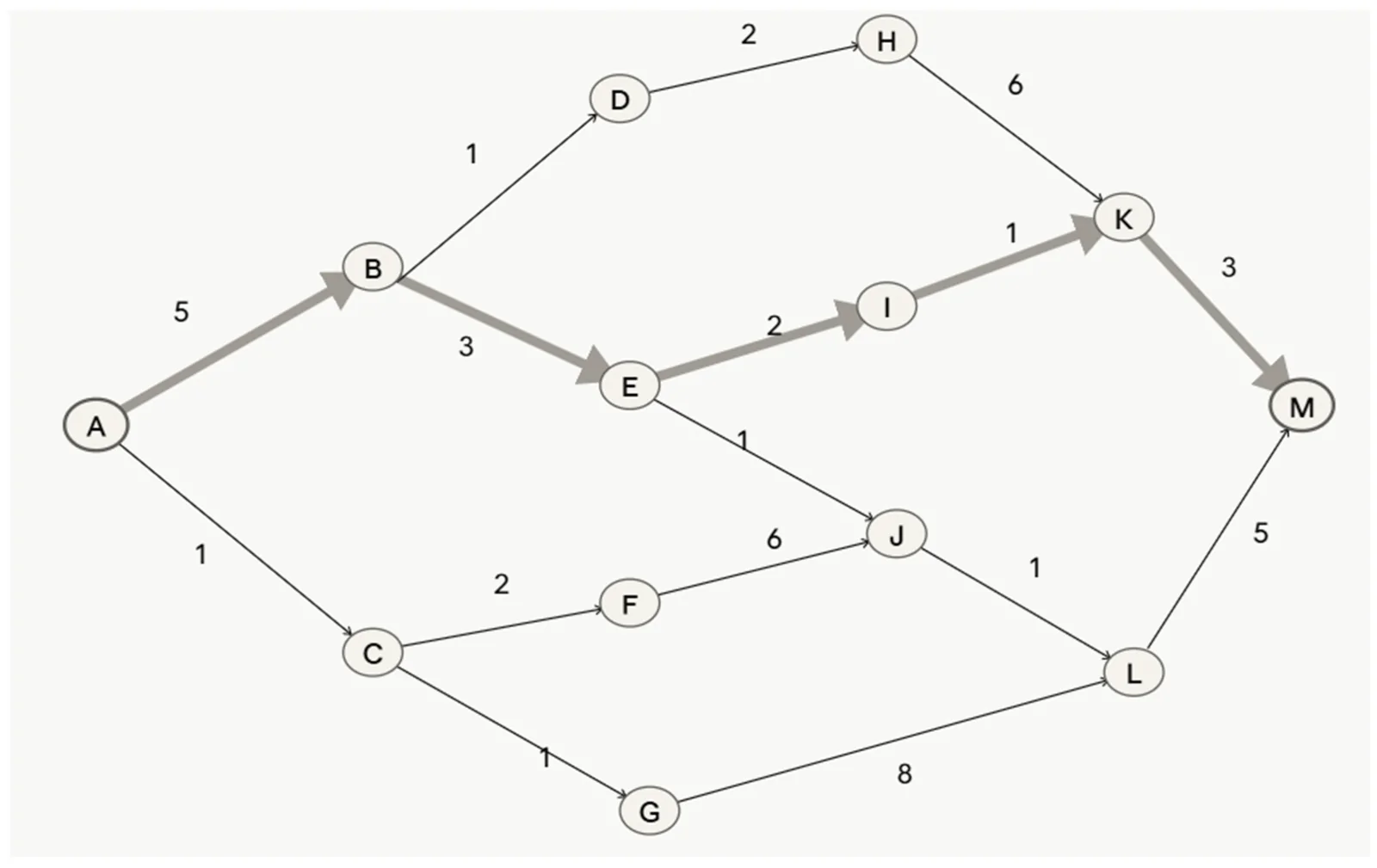
A graph with a highlighted shortest path between two vertices.

Some graph structures pose challenges for feature resolution within a graph. For instance, circular structures within a graph are difficult to distinguish in terms of orientation or vertex order, causing embeddings to appear identical even when the underlying structures are not. Geodesic-based GNNs resolve this problem by incorporating a subtree resolution step, wherein the shortest paths between vertex pairs are used to weight and aggregate pooling layers, ensuring that substructures of subtrees no longer look identical at the vertex level (Figure 10) [34]. This method has achieved state-of-the-art results on node, edge, and graph classification tasks [34] and shows considerable potential for medical imaging classification tasks due to the presence of circular structures and branching processes.

Another geodesic-based approach designed to address graph branching processes is the hyperbolic GNN, which leverages the negative curvature native to most real-world graphs [35]. Hyperbolic GNNs involve hyperbolic layers that map the graph’s manifold to a tangent space to obtain geodesic measurements [26]. Rotation and normalization operators can be incorporated into this hyperbolic mapping step. While these incarnations of hyperbolic GNNs have shown promise in recommender system problems, mapping manifolds to their tangent spaces requires high computational effort, limiting their practical applications.

### 7.10. Integrating Topology, Geometry, Radiomics, and Generative AI in Biomedical GNN Pipelines

Topology, geometry, radiomics, and generative AI contribute complementary information to biomedical GNN pipelines. Radiomics provides quantitative image-derived node, edge, or graph-level features, including intensity, texture, shape, and higher-order descriptors [6]. Topology encodes multi-scale structure, including connected components, loops, voids, higher-order relationships, and persistence-based descriptors that may reflect tumor heterogeneity, tissue architecture, vascular branching, cortical folding, or functional network organization [7,8,9,10,11,12,13,14,15,16,17,18,19,20,21,22]. Geometry adds spatial priors such as curvature, geodesic distance, equivariance, and symmetry constraints, which are relevant to anatomical surfaces, molecular structures, pathology images, vascular trees, and orientation-dependent imaging workflows [23,24,25,26,27,28,29,30,31,32,33,34,35]. Generative AI can support this pipeline by augmenting limited datasets, generating plausible graph structures, imputing missing edges, pretraining graph representations, or testing model robustness under controlled perturbations [36,37,38,39,40,41,42,43,132,133,134,135,136]. In an integrated medical imaging workflow, graph nodes may be derived from segmented lesions, anatomical structures, superpixels, cells, or radiomics clusters; edges may encode spatial adjacency, feature similarity, vascular relationships, functional connectivity, or molecular interaction; topology may summarize multi-scale connectivity and higher-order structure; geometry may preserve shape, curvature, orientation, or symmetry; and generative models may augment scarce data or synthesize graph perturbations.

## 8. Overview of GNN-Based Applications in Healthcare

### 8.1. Cancer Diagnosis

GNNs have emerged as a powerful tool in the field of cancer diagnosis, leveraging their ability to model complex relationships within biological data. Traditional machine learning approaches often struggle to capture the intricate interactions present in biomedical data, such as the relationships between different genes, proteins, and cellular pathways. GNNs, in contrast, excel at handling graph-structured data, making them particularly suited for tasks in genomics and molecular biology (Table S3) [7,8,9,10,11,12,13,14,15,16,17,18,19,20,21,22,23,24,25,26,27,28,29,30,31,32,33,34,35,113,114,115,116].

Representative application studies were selected from the structured narrative search to illustrate major biomedical domains, model families, data modalities, graph construction strategies, reported machine-learning performance measures, comparator information when available, and translational limitations rather than to provide an exhaustive meta-analysis or direct head-to-head benchmarking.

For instance, in cancer diagnosis, GNNs can analyze gene expression data, wherein nodes represent genes and edges represent interactions or co-expression relationships. By learning the underlying graph structure, GNNs can identify key biomarkers and molecular signatures associated with different cancer types, aiding in more accurate and early diagnosis.

Moreover, GNNs can integrate heterogeneous data sources—such as genetic, clinical, and imaging data—into a unified framework, enhancing the diagnostic process. Recent studies have demonstrated the effectiveness of GNNs in predicting patient outcomes, identifying potential drug targets, and classifying cancer subtypes with high accuracy. By modeling complex characteristics of cancer, GNNs have the potential to inform personalized treatment approaches. Table 1 presents a summary and comparison of GNN-based cancer diagnosis studies.

### 8.2. Diagnosis of Neurological Disorders

#### 8.2.1. Alzheimer’s Disease

GNNs offer valuable tools in diagnosing and treating Alzheimer’s disease through multiple approaches. GNNs analyze complex biological data such as genetic profiles and neuroimaging data, identifying key biomarkers and changes in brain connectivity by treating data points as nodes connected by their biological relationships. They also facilitate patient stratification, enabling personalized treatment plans by classifying patients based on disease severity or subtype. Additionally, GNNs contribute to drug discovery and repurposing by modeling molecular interactions within the brain and predicting the efficacy of potential treatments.

The study by Hernandez et al. demonstrated a multi-modal GNN approach that integrates structural MRI (sMRI) and positron emission tomography (PET) images with phenotypic data to enhance Alzheimer’s disease (AD) diagnosis. The model constructs brain networks and employs GNNs within a population graph framework to effectively leverage multi-source data [77]. Ma et al. developed an attention-guided deep GNN, utilizing local information through an attention-guided random walk to robustly handle dynamic brain networks for AD analysis [78]. Kim introduced an interpretable GNN for AD prognosis using longitudinal neuroimaging data, highlighting the model’s ability to provide biologically meaningful interpretations of neuroanatomical data [79].

Song et al. proposed an auto-metric GNN for AD diagnosis and progression prediction, employing a metric-based meta-learning strategy to facilitate inductive learning and effective multimodal data integration [80]. Song et al. focused on using graph convolutional neural networks for staging and classification of AD across a disease spectrum, demonstrating superior performance over traditional models using structural connectivity graphs [81]. Zhang et al. echoed the multi-modal GNN framework by Hernandez, integrating sMRI and PET data for enhanced AD diagnosis predictions [82]. Klepl et al. applied GNNs to classify EEG brain graphs, using various functional connectivity measures to demonstrate the superior performance of GNNs over conventional models in diagnosing AD [83]. Sampathkumar et al. developed ADiag, employing a GraphSAGE network and dense differentiable pooling to analyze cortical thickness for AD diagnosis, showcasing robust diagnostic accuracy [84]. Gao et al. utilized a GNN model to predict brain age from resting-state fMRI data, demonstrating the potential of GNNs in predicting accelerated brain aging associated with AD [85]. Table 2 presents a comparison of GNNs for Alzheimer’s disease diagnosis.

#### 8.2.2. Dementia Analysis

GNNs are highly effective in dementia analysis due to their ability to handle the complex and interconnected data typical of brain imaging studies. These networks excel in modeling both functional and structural connectivity within the brain, making them suitable for analyzing MRI, fMRI, or EEG data. GNNs can extract meaningful features from high-dimensional brain data, classify stages of dementia, and predict disease progression by analyzing changes over time. They also integrate multimodal data sources, including clinical and genetic information, enhancing diagnostic accuracy and treatment planning. Additionally, GNNs provide interpretability by identifying key biomarkers and brain regions involved in the disease, crucial for understanding dementia’s mechanisms and developing personalized therapeutic strategies.

Wang et al. (2022) introduced a GNN that captures global relationships between brain regions using rs-fMRI data [86]. The model combines self-attention structures for diagnosis with feature selection for noise reduction and improved interpretability, demonstrating enhanced diagnostic performance on multiple neurological diseases. Cao et al. (2024) developed a directed structure learning GNN (DSL-GNN) for differentiating Alzheimer’s disease (AD), Parkinson’s disease (PD), and healthy controls using effective brain connectivity (EBC) and power spectrum density (PSD) features, demonstrating high accuracy in discrimination tasks [87]. Hasan et al. (2024) presented a novel CNN-GCN architecture for staging dementia, attaining perfect accuracy on MRI scans from the Alzheimer’s Disease Neuroimaging Initiative, highlighting its potential for detailed dementia stage classification [88]. de Haan et al. (2009) applied graph theory to analyze EEG-based functional brain networks, finding deviations from optimal network structures in Alzheimer’s patients and supporting the disconnection hypothesis [89]. Hu et al. (2023) utilized a variationally regularized encoder–decoder GNN (VGNN) with claims data for Alzheimer’s disease risk prediction, outperforming traditional risk prediction models [90]. Kim et al. (2021) [79] proposed an interpretable GNN for AD prognostic prediction using longitudinal neuroimaging data, outperforming several standard machine learning approaches [109]. Table 3 summarizes GNN-based dementia analysis frameworks.

## 9. Cardiovascular Modeling and Simulation

GNNs have emerged as a powerful tool in cardiovascular modeling and simulation due to their ability to efficiently handle complex graph-structured data, such as vascular networks. Pegolotti et al. (2024) developed a one-dimensional reduced-order model using a GNN trained on three-dimensional hemodynamic simulation data to predict blood flow dynamics, achieving errors below 3% for pressure and flow rate and outperforming traditional physics-based models [91]. De Chou (2024) integrated DL with numerical simulations of partial differential equations to create a physics-informed GNN for myocardial perfusion simulation, demonstrating promising generalization capabilities for non-invasive diagnostic tools in clinical settings [92]. Yao (2024) introduced Image2Flow, a hybrid model combining image and graph convolutional neural networks to generate patient-specific volume meshes and estimate CFD flow fields from 3D cardiac MRI data, achieving excellent segmentation accuracy and completing tasks significantly faster than traditional manual methods [93].

### 9.1. Cardiac Health and Myocardial Infarction Detection

Abisoye (2024) combined CNNs and GNNs to enhance the detection of myocardial infarction (MI) from long-term ECG data, significantly improving the performance of MI detection and achieving high precision, recall, and accuracy [94]. Kutluana (2024) explored the use of visibility graphs to convert ECG signals into graph representations classified using ResNet and Inception models, achieving superior classification results on the PTB-XL dataset [95]. EPMoghaddam (2023) introduced a method for classifying cardiac arrhythmias by converting ECG heartbeats into graphs using the visibility graph technique and applying a graph convolutional neural network (GCN), demonstrating high accuracy, precision, and recall in classifying arrhythmias from the MIT-BIH arrhythmia database [96].

### 9.2. Chronic Disease and Coronary Artery Disease

Rico (2024) proposed a GNN model with Laplacian regularization to analyze the relationships between multiple chronic conditions and patient-specific risk factors, outperforming baseline GNNs in predicting the co-occurrence of chronic diseases [97]. Zhao (2024) presented a multi-graph matching algorithm (MGM) for semantic labeling of coronary artery segments in coronary angiography, achieving high accuracy in labeling arterial segments, aiding in coronary artery analysis and CAD diagnosis [98]. Lin (2023) developed a domain-adaptive multichannel GCN (DAMGCN) for transferring knowledge across different coronary heart disease (CHD) datasets, achieving high performance across multiple CHD datasets through a two-channel GCN with an attention mechanism and a domain adversarial module [99]. Zhao (2023) introduced the edge attention graph matching network (EAGMN) for semantic labeling of coronary artery segments, achieving high accuracy in semantic labeling and providing a novel tool for CAD diagnosis [100].

### 9.3. Other Applications in Cardiovascular Health

Zhang (2023) [82] proposed a spatial-temporal residual GCN for diagnosing cardiovascular diseases from multi-lead ECG signals, outperforming existing algorithms in terms of F1 scores on the PTB-XL and Chapman databases [101]. Walczak (2023) introduced a deep-learning-based method for reconstructing 3D surface models of the mitral valve from 3D TEE images, combining CNN and GNN for mesh deformation and achieving high accuracy in mitral valve reconstruction, aiding in surgical planning [102]. Duong (2023) investigated the use of GNNs for ECG classification, achieving human-level accuracy in ECG classification as a non-invasive pre-screening tool [103]. Table 4 presents a comprehensive comparison of studies on GNNs in medical and cardiovascular applications.

### 9.4. Cross-Model Analytical Considerations for Biomedical GNN Applications

Although Table 1, Table 2, Table 3 and Table 4 summarize representative GNN-based biomedical studies by application area, model type, data source, performance, and limitations, study-level comparisons alone may obscure broader translational differences among GNN model families. Therefore, Table 5 provides a cross-model synthesis of analytical criteria that are particularly relevant to medical imaging and biomedical implementation, including data modality, computational cost, scalability, interpretability, clinical suitability, and implementation limitations. These criteria are important because biomedical GNN performance depends not only on reported accuracy or AUC, but also on how the graph is constructed, how well the model scales to large or multimodal datasets, whether its predictions can be interpreted clinically, and whether it can be validated across institutions, scanners, protocols, and patient populations [37,38,39,40,41,42,43,59,60,61,62,63,64,65,66,67,68,69,70,71,72,73,74,75,76,77,78,79,80,81,82,83,84,85,86,87,88,89,90,91,92,93,94,95,96,97,98,99,100,101,102,103,104,105,132,133,134,135,136,140,141,150].

The application tables summarize the study-level findings as reported by the original articles, whereas Table 5 provides a higher-level methodological comparison across GNN model families. This distinction is important because biomedical GNN studies differ substantially in dataset size, data modality, graph construction, validation strategy, baseline comparators, and reported performance metrics. Therefore, model performance should be interpreted in the context of each study’s task and validation design rather than as a direct ranking across applications. Future biomedical GNN studies should more consistently report dataset characteristics, graph construction details, training and validation design, baseline comparators, computational requirements, and clinically relevant performance metrics.

### 9.5. Key Takeaways and Future Directions

The reviewed literature suggests that GNNs are most useful in biomedical imaging when graph construction is biologically or anatomically meaningful and when model evaluation extends beyond internal performance metrics. Reported results should be interpreted in the context of node and edge definitions, dataset size, validation design, comparator models, and clinical endpoint, rather than as direct comparisons across heterogeneous studies. Topology-aware, geometry-aware, hybrid graph–transformer, and generative graph methods offer promising directions for modeling multi-scale structure, symmetry, long-range dependencies, and low-label learning, but they also increase computational complexity and validation requirements [7,8,9,10,11,12,13,14,15,16,17,18,19,20,21,22,23,24,25,26,27,28,29,30,31,32,33,34,35,37,38,39,40,41,42,43,132,133,134,135,136].

Future research should emphasize standardized graph construction, transparent reporting of preprocessing and validation design, external testing across institutions and imaging protocols, interpretability, calibration, uncertainty estimation, fairness assessment, and robustness to missing or noisy graph features. For more complex architectures, including hybrid graph–transformer and generative graph models, studies should demonstrate incremental clinical value over simpler GNN baselines. Ultimately, prospective evaluation is needed to determine whether GNN-based tools improve radiologist workflow, diagnostic accuracy, risk stratification, treatment planning, or patient outcomes.

Morse functions may provide a useful future direction for topology-aware GNNs in medical imaging. A Morse function is a smooth real-valued function on a manifold with non-degenerate critical points, allowing topological structure to be studied through scalar-value changes. In imaging, analogous functions could be defined from intensity, color, texture, probability maps, distance transforms, or radiomic gradients to identify critical points where boundaries or subtle features change. Such preprocessing may help define graph nodes, edges, or regions of interest in small, diffuse, or poorly marginated structures, such as infiltrative tumors. Although this approach remains exploratory for medical imaging GNNs, Morse-theoretic preprocessing has shown promise for feature definition before GNN modeling in irregular spatial domains [151].

To improve practical usability for readers, representative open-source software resources for implementing GNNs, topology-aware models, geometry-aware models, equivariant GNNs, sheaf neural networks, radiomics extraction, and medical-imaging preprocessing are provided in Table S4. These resources are intended as implementation starting points rather than endorsed clinical software, and users should verify software maintenance status, licensing, dependencies, and suitability for their own datasets before use.

## 10. Open Challenges

As summarized in Table 5 and Section 9.5, the major barriers to biomedical GNN translation extend beyond predictive performance and include graph construction variability, dataset heterogeneity, computational cost, scalability, interpretability, limited external validation, and uncertain clinical utility. These challenges recur across oncologic, neurologic, cardiovascular, and multimodal biomedical applications [59,60,61,62,63,64,65,66,67,68,69,70,71,72,73,74,75,76,77,78,79,80,81,82,83,84,85,86,87,88,89,90,91,92,93,94,95,96,97,98,99,100,101,102,103,104,105].

Moreover, processing complexities with spatial data and protein–protein interaction networks [74,75] demonstrate the difficulty in capturing complex biological interactions. Issues such as limited scope and drug specificity [67] further emphasize the need for models that can translate across different therapeutic areas. The requirement for further verification in varied clinical or experimental settings [76] underlines ongoing concerns about the reliability and ethical implications of deploying AI models in healthcare.

A notable challenge is the complexity of integrating heterogeneous data types such as sMRI, PET, and phenotypic data, highlighted in studies by Hernandez et al. [77] and Zhang et al. [82]. Additionally, Ma et al. [78] point out the sensitivity of these models to graph noise, suggesting a need for robust mechanisms to manage noise and ensure data integrity. The requirement for extensive computational resources, as reported by Kim et al. [79], restricts the scalability and practical deployment of GNNs in clinical settings. Performance variability across different stages of Alzheimer’s disease, noted by Song et al. (2019) [55], indicates that current models may not perform consistently across the disease spectrum, calling for the development of adaptive models that can accurately reflect disease progression.

Challenges in integrating phenotypic data and inconsistencies in feature extraction methods—such as those involving different functional connectivity measures from EEG signals (Klepl et al. [83])—indicate a need for standardized and optimized feature extraction methods. The reliance on visual observation for MRI analysis (Sampathkumar et al. [84]) introduces potential bias, pointing toward a requirement for more objective and automated diagnostic processes. Lastly, the comparatively lower accuracy of certain GNN applications relative to traditional methods (Gao et al. [85]) highlights the need for advances in GNN architectures or the development of hybrid models that combine the strengths of various approaches.

The growing adoption of GNNs in cardiovascular modeling and simulation reveals significant challenges that must be addressed to fully exploit their potential. A recurring theme is the need for large and diverse datasets to train these networks effectively, as demonstrated by Pegolotti et al. [91] and Duong [103]. The complexity of integrating and processing diverse data types—from ECG signals to invasive coronary angiography videos—poses another critical challenge. The high computational demands and algorithmic complexity, such as those reported by Lin [99] and Zhao [105], could hinder practical deployment in clinical settings. These challenges underscore the need for more robust, scalable, and generalizable GNN models capable of handling the complexities of cardiovascular data while ensuring accuracy and efficiency in real-world medical applications.

## 11. Conclusions

GNNs are increasingly used in cardiovascular modeling and may improve diagnostic and treatment approaches. However, several challenges currently hinder the realization of their full potential. The necessity for extensive, diverse datasets is evident, as GNNs require substantial training data to maintain accuracy in dynamic applications such as predicting blood flow dynamics or classifying ECG signals. Furthermore, the integration and processing of heterogeneous types of data, such as imaging, signals, and clinical data, introduce significant complexities. High computational and technical requirements limit the use of these models in clinical settings. These challenges suggest a crucial need for advancements in GNN methodologies, aiming for models that are robust, scalable, and generalizable across various cardiovascular conditions.

Methods that incorporate topology and geometry have improved GNN performance in certain classification tasks, including medical imaging. Simplicial neural networks in particular offer a fruitful path forward in developing the next generation of medical imaging GNNs, as they provide many ways to pool neighborhood results in a multiscale fashion and can reduce computational complexity while still outperforming specialized GNNs on medical imaging tasks. There are many other avenues for modifying the calculations of HL-HGAT, adapting frameworks to run on quantum computers, and introducing new types of spectral and spatial pooling strategies based on tools that extend graph methods to simplicial complexes.

From a practical medical imaging perspective, no single GNN architecture is universally optimal; the preferred model depends on graph construction, clinical endpoint, dataset size, and interpretability requirements. For structured imaging graphs, such as region-adjacency graphs, brain-connectivity networks, molecular interaction graphs, or patient-similarity graphs, conventional GCN, GraphSAGE, MPNN, or attention-based GNN models remain appropriate first-line baselines because they are easier to train, interpret, and externally validate [113,114,115,116,117,118,119,120,121,122,123,124,125,126,127,128,129,130,131,140,141,142,143]. When the disease phenotype depends on multi-scale shape or tissue organization, persistent-homology-enhanced GNNs may be especially useful because connected components, loops, cavities, and persistence intervals can be related to clinically meaningful imaging patterns such as tumor heterogeneity, necrosis, glandular architecture, vascular branching, or cortical folding [7,8,9,10,11,12,13,14,15,16,17]. Sheaf neural networks offer a more flexible but less clinically mature alternative because they permit functions to vary across graph, simplicial, or cellular structures that share boundaries [21]. Recent Hilbert-bundle and cellular-sheaf formulations provide theoretical guarantees for learning on irregular domains, time-varying signals, and distribution-valued data, suggesting potential adaptability to real-world imaging tasks [152]. Nevertheless, because persistence-based approaches are more interpretable and easier to verify clinically, we recommend that future medical imaging studies begin with transparent graph construction and simpler GNN baselines, then introduce sheaf, equivariant, hybrid graph–transformer, or generative graph models only when they demonstrate incremental value through ablation analysis, external validation, calibration, and clinical utility assessment.

## Figures and Tables

**Table 1:** Summary and comparison of GNN-based cancer diagnosis studies.

Ref.	Model Used	Application	Data Used	Reported Performance/Main Finding	Limitation
[59]	Geometric GNN (GGNN)	Cancer prognosis prediction	Multi-omics data from CoMMpass study and TCGA	Improved prediction relative to reported comparator models	High-dimensional, low-sample size problem
[60]	Multi-omics GNN framework	Cancer subtype classification	TCGA Pan-cancer and BRCA datasets	High accuracy, F1 score, precision, and recall	Limited to binary omic connections
[61]	Causality-driven GNN	Early diagnosis of pancreatic cancer	Multi-center dataset	High stability and generalizability	Specific to non-contrast CT scans
[62]	LAGProg (Local Augmented GNN)	Cancer prognosis prediction	Multi-omics data from TCGA	Improved C-index values by 8.5%	Limited neighboring gene data in networks
[63]	Explainable Multilayer GNN (EMGNN)	Identification of cancer genes	Pan-cancer multi-omics data	7.15% improvement in AUC	Variability in network predictions
[64]	GGraphSAGE	Prediction of cancer driver genes	Multiomics data, including PPI networks	Improved performance compared with reported state-of-the-art comparator models	Specific to cancer types, not pan-cancer
[65]	ICInet (DeepOmix-ICI)	Prediction of immune therapy response	Data from 600 ICI-treated patients	AUC = 0.85	Comparator biomarker models showed lower predictive performance than the integrated DeepOmix-ICI framework, but the relative contribution of each individual biomarker class requires further clarification and external validation
[66]	GAMB-GNN	Cancer classification using microarray data	Public microarray datasets	Accuracy and F1-score improved significantly	Redundancy in feature selection
[67]	GEFA model (GNN-based)	Drug repurposing for COVID-19	Data from DrugBank and PubChem	Identification of alternative treatments	Drug-repurposing analysis was restricted primarily to kinase inhibitors, limiting generalizability to other drug classes, targets, and therapeutic mechanisms
[68]	GNN	Breast cancer tumor grading	Public histopathology datasets	Performance metric not clearly specified in source article	Model emphasized local histopathologic features and may incompletely capture global tissue architecture or slide-level spatial organization
[69]	SLGNN (GNN)	Synthetic lethality prediction	Gene-related knowledge graphs	Improved performance compared with reported baseline models	Need for deeper SL mechanism understanding
[70]	4-layer GCN	Survival prediction for cancer patients	Whole slide images	C-index values: 0.57, 0.64	Limited to gastric and colon adenocarcinoma
[71]	MPK-GNN (GNN)	Cancer molecular subtype classification	Multi-omics data	Improved performance compared with comparator models reported in the source study	Requires integration of heterogeneous multi-omics features and graph priors, which may increase preprocessing complexity and reduce reproducibility across datasets
[72]	HAGNN (GNN)	Gene subset selection for disease classification	Microarray data	Improved performance compared with comparator models reported in the source study	High computational demand may limit scalability for large microarray or multi-omics datasets without optimized hardware or sampling strategies
[73]	Graph Convolutional Network (GCN)	Pancreatic tumor detection	Whole slide images	F1 score: 0.85	Validation in clinical settings needed
[74]	STGNNks (GNN)	Spatial transcriptomics analysis	10x Genomics Visium datasets	Highest clustering performance among reported comparator methods	Requires specialized preprocessing of spatial transcriptomics data, including spatial-neighborhood definition and normalization across tissue sections
[75]	SMG (GNN)	Cancer genomics	Protein-protein interaction networks	Improved performance compared with baseline models reported in the source study	Performance may be constrained by limited labeled cancer-genomics data and dependence on the completeness of protein–protein interaction networks
[76]	AttenSyn (GNN)	Synergistic drug combination prediction	Molecular graphs of drugs	Improved performance compared with baseline models reported in the source study	Requires validation across additional cancer cell lines, drug combinations, and experimental settings to assess generalizability

Reported performance values and main findings are summarized as described in the cited studies. Because the reviewed studies vary in task definition, dataset size, validation design, graph construction strategy, comparator model, and reported metric, values should not be interpreted as direct head-to-head comparisons across models. Comparator methods are specified where reported by the original study.

**Table 2:** Comparison of studies on GNNs for Alzheimer’s disease diagnosis. Limitations marked with † are explicitly stated in the cited paper; remaining entries reflect our own assessment based on a critical reading of the original work.

Ref.	Model Used	Application	Data Used	Reported Performance/Main Finding	Limitation †
[77]	Multi-modal GNN	AD diagnosis	sMRI, PET, phenotypic data	Improved performance with multi-modal integration	Limited by data heterogeneity and integration complexity
[78]	AGDGN with AGRW module	Longitudinal AD data analysis	ADNI dataset	Effective in identifying informative brain regions	Sensitivity to graph noise
[79]	Interpretable GNN	AD prognostic prediction	Longitudinal neuroimaging data	Higher accuracy than DNN and SVM comparator models	Requires extensive computational resources
[80]	AMGNN	AD diagnosis and progression prediction	TADPOLE dataset	Accuracies of 94.44% for AD diagnosis, 87.50% for MCI conversion	Performance can depend on meta-task configuration
[81]	GCNN	AD classification and staging	Diffusion tensor imaging data	Higher performance than SVM, with improved classification across later disease stages	Classification performance varies across AD spectrum
[82]	Multi-modal GNN	AD diagnosis	sMRI, PET, phenotypic data	Improved diagnostic performance	Phenotypic data integration challenges
[83]	GNN with various FC measures	Automated AD diagnosis	EEG signals	AUC of 0.984, 92% accuracy	Inconsistency across different FC measures
[84]	GraphSAGE network with DDP	AD diagnosis	MRI cortical thickness data	Robust accuracy of 83%	Reliance on visual observation for MRI analysis
[85]	GNN with attention mechanism	Brain age prediction	rs-fMRI data	Prediction MAE of 5.92 years	Lower accuracy compared to structural MRI-based studies

Reported performance values and main findings are summarized as described in the cited studies. Because the reviewed studies vary in task definition, dataset size, validation design, graph construction strategy, comparator model, and reported metric, values should not be interpreted as direct head-to-head comparisons across models. Comparator methods are specified where reported by the original study.

**Table 3:** Comparison of GNN models for neurodegenerative disease diagnosis.

Ref.	Model Used	Application	Data Used	Reported Performance/Main Finding	Limitation
[86]	Self-attention GNN	Brain disease diagnosis	rs-fMRI	High diagnostic performance	Interpretability issues
[87]	DSL-GNN	Dementia diagnosis	EBC estimations, PSD features	94.0% to 97.4% accuracy	Complexity in handling directional information
[88]	CNN-GCN	Dementia stage prediction	MRI scans	Up to 100% accuracy	High model complexity
[89]	Graph theory	Network dysfunction analysis in dementia	EEG recordings	Decreased connectivity in AD	Limited to EEG analysis
[90]	VGNN	ADRD risk prediction	Claims data	10% better AUC than baselines	Requires complex data preprocessing
[79]	Interpretable GNN	AD prognostic prediction	Longitudinal neuroimaging data	Outperforms DNN, SVM	High computational demand

Reported performance values and main findings are summarized as described in the cited studies. Because the reviewed studies vary in task definition, dataset size, validation design, graph construction strategy, comparator model, and reported metric, values should not be interpreted as direct head-to-head comparisons across models. Comparator methods are specified where reported by the original study.

**Table 4:** Comparison of studies on GNNs in medical and cardiovascular applications.

Ref.	Model Used	Application	Data Used	Reported Performance/Main Finding	Limitation
[91]	Graph neural network	Blood flow dynamics simulation	Three-dimensional hemodynamic simulation data	Errors below 3% for pressure and flow rate	Requires adequate training data
[94]	CNN with GNN	Myocardial infarction detection	Long-term ECG data	F1 score of 99.58%, Precision of 99.5%, Accuracy of 99.72%	None specified
[92]	Physics-informed GNN	Myocardial perfusion simulation	3D synthetic and patient CT datasets	Promising generalization capabilities	Limited details on specific performance metrics
[97]	Laplacian regularized GNN	Analysis of chronic diseases	Data from Cameron County Hispanic Cohort	Average accuracy of 89%	Performance declines with more conditions
[98]	Multi-graph graph matching algorithm	Coronary artery semantic labeling	ICA videos	Accuracy of 0.9471	Focus on morphology could overlook functional insights
[95]	Visibility graphs with ResNet and Inception	ECG signal classification	PTB-XL dataset	AUC score of 93.46%	High dimensionality reduction required
[93]	Hybrid image and graph CNN	CFD flow field estimation from cardiac MRI	3D cardiac MRI data	Median Dice score of 0.9	High performance dependency on accurate mesh creation
[104]	LSTM with visibility graph	Heart rate variability analysis during meditation	Physionet database	Accuracy of 99.25%	Specific to meditation context
[103]	GNN	ECG classification for CVDs	Data from MITBIH and PTB	Accuracy of 1.0	Requires large datasets for training
[99]	Adversarial domain-adaptive GCN	Cross-domain CHD knowledge transfer	Various CHD datasets	Highest reported performance across three CHD datasets compared with study baselines	Complex model structure
[101]	Spatial-temporal residual GCN	Cardiovascular disease diagnosis	PTB-XL and Chapman databases	Increases in F1 by 5.85% and 6.80%	Over-smoothing and fitting issues
[100]	Edge attention graph matching network	Coronary artery semantic labeling	ICA images	Weighted F1-score of 0.8643	High complexity in graph matching
[105]	Association graph-based graph matching network	Coronary arterial semantic labeling	ICA images	Average F1-score of 0.8262	High algorithmic complexity
[102]	CNN with GNN	3D Mitral valve reconstruction	3D TEE images	Average distance of 1.1 mm	Challenging in capturing fast-moving leaflets
[96]	Graph convolutional network	Cardiac arrhythmia classification	MIT-BIH arrhythmia database	Average accuracy of 98.16%	Specific to arrhythmia detection

Reported performance values and main findings are summarized as described in the cited studies. Because the reviewed studies vary in task definition, dataset size, validation design, graph construction strategy, comparator model, and reported metric, values should not be interpreted as direct head-to-head comparisons across models. Comparator methods are specified where reported by the original study.

**Table 5:** Analytical criteria for major GNN model families in biomedical applications.

Model Family	Data Modality	Cost/Scalability	Interpretability	Clinical Suitability	Main Implementation Limitation	References
Spectral GNNs/GCNs	Brain networks, radiomics graphs, molecular graphs, region-adjacency graphs	Efficient for first-order GCNs; full spectral methods less scalable	Moderate	Useful for structured graphs with stable topology	Over-smoothing; limited transferability across heterogeneous graphs	[113,114,115,116,138,140,141]
Spatial message-passing GNNs	Biological networks, cell graphs, patient graphs, region-adjacency graphs	Scalable with sampling	Moderate	Useful when local relationships are clinically meaningful	Sensitive to graph construction and neighborhood definition	[117,119,120,121,140,141,142,143]
Attention-based GNNs	Multimodal graphs, pathology cell graphs, molecular graphs, dynamic brain networks	More expensive with dense graphs or many attention heads	Moderate to high	Useful when node/edge/modal relevance varies	Attention weights are not always faithful explanations	[86,100,122,125,126,127,128,129,130,131]
Graph–transformer hybrids	Multimodal graphs, brain networks, tractography, radiomics graphs	High unless sparse/local-global attention is used	Moderate	Promising for long-range and multiscale dependencies	High memory burden; risk of overfitting small datasets	[127,130,131,132,133,134,135,136,150]
Topology-aware GNNs	Tumor graphs, tissue architecture, vascular graphs, cortical surfaces	Moderate to high	Potentially high	Useful for shape, connectivity, loops, cavities, and branching	Requires specialized topological preprocessing	[7,8,9,10,11,12,13,14,15,16,17,18,19,20,21,22]
Geometry-aware/equivariant GNNs	Molecular graphs, anatomical meshes, pathology images, vascular trees	Moderate to high	Moderate to high	Useful when symmetry, shape, or curvature is clinically meaningful	Requires correct symmetry/geometric prior	[23,24,25,26,27,28,29,30,31,32,33,34,35]
Temporal/spatiotemporal GNNs	ECG, longitudinal imaging, dynamic connectivity, patient trajectories	Moderate to high	Moderate	Useful for monitoring and disease progression	Sensitive to missingness and irregular sampling	[78,86,101,131,142]
Generative graph models	Molecular graphs, cell graphs, brain networks, radiomics graphs	Variable; often high for large graphs	Variable	Useful for augmentation and low-label settings	Synthetic-to-real domain shift; biologically implausible graphs	[37,38,39,40,41,42,43,55,139]
Physics-informed/domain-adaptive GNNs	Hemodynamics, myocardial perfusion, coronary graphs, CFD meshes	High during training; variable at inference	Moderate to high	Useful for constrained clinical modeling	Requires domain assumptions, simulation data, or adaptation design	[91,92,93,97,98,99,100]

## Data Availability

Data derived from public domain resources.

## Supplementary Materials

The following supporting information can be downloaded at: https://www.mdpi.com/article/10.3390/bioengineering13060638/s1. Table S1. Taxonomy of GNN model families and graph-learning strategies relevant to biomedical imaging analysis. Table S2. Training and scalability considerations for biomedical GNNs. Table S3. Mathematical operators and representation-learning roles in topology- and geometry-aware GNNs. Table S4. Representative open-source software resources for GNN-based biomedical imaging and graph-learning workflows.

## Abbreviations

The following abbreviations are used in this manuscript:

DL	Deep Learning
AI	Artificial Intelligence
GANs	Generative Adversarial Networks
LLMs	Large Language Models
GATs	Graph Attention Networks
MPNNs	Message Passing Neural Networks
PH	Persistent Homology
TDA	Topological Data Analysis

## References

[1] Habchi Y., Himeur Y., Kheddar H., Boukabou A., Atalla S., Chouchane A., Ouamane A., Mansoor W. (2023). AI in Thyroid Cancer Diagnosis: Techniques, Trends, and Future Directions. Systems.

[2] Dixit S., Bohre K., Singh Y., Himeur Y., Mansoor W., Atalla S., Srinivasan K. (2023). A Comprehensive Review on AI-Enabled Models for Parkinson’s Disease Diagnosis. Electronics.

[3] D’Souza N.S., Wang H., Giovannini A., Foncubierta-Rodriguez A., Beck K.L., Boyko O., Syeda-Mahmood T.F. (2024). Fusing Modalities by Multiplexed Graph Neural Networks for Outcome Prediction from Medical Data and Beyond. Med. Image Anal..

[4] Waikhom L., Patgiri R. (2021). Graph Neural Networks: Methods, Applications, and Opportunities. arXiv.

[5] Khemani B., Patil S., Kotecha K., Tanwar S. (2024). A Review of Graph Neural Networks: Concepts, Architectures, Techniques, Challenges, Datasets, Applications, and Future Directions. J. Big Data.

[6] Zhang X., Zhang X., Yang G., Zhang L., Chen Z., Wang J., Zhang Y., Chen W., Wang X., Li X. (2022). Deep Learning with Radiomics for Disease Diagnosis and Treatment: Challenges and Potential. Front. Oncol..

[7] Ghrist R. (2008). Barcodes: The Persistent Topology of Data. Bull. Am. Math. Soc..

[8] Zomorodian A., Carlsson G. Computing Persistent Homology. Proceedings of the Twentieth Annual Symposium on Computational Geometry.

[9] Edelsbrunner H., Harer J., Goodman J.E., Pach J., Pollack R. (2008). Persistent Homology—A Survey. Surveys on Discrete and Computational Geometry: Twenty Years Later.

[10] Xu K., Hu W., Leskovec J., Jegelka S. How Powerful Are Graph Neural Networks?. Proceedings of the International Conference on Learning Representations.

[11] Chen Z., Chen L., Villar S., Bruna J. (2020). Can Graph Neural Networks Count Substructures?. Adv. Neural Inf. Process. Syst..

[12] Horn M., De Brouwer E., Moor M., Moreau Y., Rieck B., Borgwardt K. Topological Graph Neural Networks. Proceedings of the International Conference on Learning Representations.

[13] Bouritsas G., Frasca F., Zafeiriou S.P., Bronstein M.M. (2023). Improving Graph Neural Network Expressivity via Subgraph Isomorphism Counting. IEEE Trans. Pattern Anal. Mach. Intell..

[14] Hofer C., Kwitt R., Niethammer M., Uhl A. Graph Filtration Learning. Proceedings of the International Conference on Machine Learning.

[15] Chen Y., Coskunuzer B., Gel Y. (2021). Topological Relational Learning on Graphs. Adv. Neural Inf. Process. Syst..

[16] Ye X., Sun F., Xiang S. (2023). TREPH: A Plug-In Topological Layer for Graph Neural Networks. Entropy.

[17] Wen T., Chen E., Chen Y. (2024). Tensor-View Topological Graph Neural Network. arXiv.

[18] Ebli S., Defferrard M., Spreemann G. (2020). Simplicial Neural Networks. arXiv.

[19] Huang J., Chen Q., Zhu P., Bian Y.-J., Chen N., Chung M.C., Qiu A. (2025). HL-HGAT: Heterogeneous Graph Attention Network via Hodge-Laplacian Operator. IEEE Trans. Pattern Anal. Mach. Intell..

[20] Hajij M., Istvan K., Zamzmi G. (2020). Cell Complex Neural Networks. arXiv.

[21] Hansen J., Gebhart T. (2020). Sheaf Neural Networks. arXiv.

[22] Shen C., Liu X., Luo J., Xia K. (2023). Torsion Graph Neural Networks. arXiv.

[23] Ye Z., Liu K.S., Ma T., Gao J., Chen C. Curvature Graph Network. Proceedings of the International Conference on Learning Representations.

[24] Li H., Cao J., Zhu J., Liu Y., Zhu Q., Wu G. (2022). Curvature Graph Neural Network. Inf. Sci..

[25] Finzi M., Stanton S., Izmailov P., Wilson A.G. Generalizing Convolutional Neural Networks for Equivariance to Lie Groups on Arbitrary Continuous Data. Proceedings of the 37th International Conference on Machine Learning.

[26] Yang J., Walters R., Dehmamy N., Yu R. Generative Adversarial Symmetry Discovery. Proceedings of the 40th International Conference on Machine Learning.

[27] Veeling B.S., Linmans J., Winkens J., Cohen T., Welling M. (2018). Rotation Equivariant CNNs for Digital Pathology. Medical Image Computing and Computer Assisted Intervention—MICCAI 2018, Granada, Spain, 16–20 September 2018.

[28] Kumar H., Parada-Mayorga A., Ribeiro A. (2024). Lie Group Algebra Convolutional Filters. IEEE Trans. Signal Process..

[29] Shutty N., Wierzynski C. Computing Representations for Lie Algebraic Networks. Proceedings of the NeurIPS 2023 Workshop.

[30] Cremer J., Medrano Sandonas L., Tkatchenko A., Clevert D.-A., De Fabritiis G. (2023). Equivariant Graph Neural Networks for Toxicity Prediction. Chem. Res. Toxicol..

[31] Yang X., Jia X., Gong D., Yan D., Li Z., Liu W. LARNet: Lie Algebra Residual Network for Face Recognition. Proceedings of the 38th International Conference on Machine Learning.

[32] Satorras V.G., Hoogeboom E., Welling M. E(n) Equivariant Graph Neural Networks. Proceedings of the 38th International Conference on Machine Learning.

[33] Dehmamy N., Walters R., Liu Y., Wang D., Yu R. (2021). Automatic Symmetry Discovery with Lie Algebra Convolutional Network. Adv. Neural Inf. Process. Syst..

[34] Kong L., Chen Y., Zhang M. (2022). Geodesic GNN for Efficient Graph Representation Learning. Adv. Neural Inf. Process. Syst..

[35] Liu Q., Nickel M., Kiela D. (2019). Hyperbolic Graph Neural Networks. arXiv.

[36] Patel H., Farrelly C., Hathaway Q.A., Rozenblit J.Z., Deepa D., Singh Y., Chaudhary A., Himeur Y., Mansoor W., Atalla S. (2023). Topology-Aware GAN (TopoGAN): Transforming Medical Imaging Advances. Proceedings of the 2023 Tenth International Conference on Social Networks Analysis, Management and Security, Abu Dhabi, United Arab Emirates, 21–24 November 2023.

[37] Kipf T.N., Welling M. (2016). Variational Graph Auto-Encoders. arXiv.

[38] Simonovsky M., Komodakis N. (2018). GraphVAE: Towards Generation of Small Graphs Using Variational Autoencoders. arXiv.

[39] You J., Ying R., Ren X., Hamilton W.L., Leskovec J. GraphRNN: Generating Realistic Graphs with Deep Auto-Regressive Models. Proceedings of the 35th International Conference on Machine Learning.

[40] De Cao N., Kipf T. MolGAN: An Implicit Generative Model for Small Molecular Graphs. Proceedings of the ICML 2018 Workshop on Theoretical Foundations and Applications of Deep Generative Models.

[41] Vignac C., Krawczuk I., Siraudin A., Wang B., Cevher V., Frossard P. DiGress: Discrete Denoising Diffusion for Graph Generation. Proceedings of the International Conference on Learning Representations.

[42] Zhang M., Qamar M., Kang T., Jung Y., Zhang C., Bae S.-H., Zhang C. (2023). A Survey on Graph Diffusion Models: Generative AI in Science for Molecule, Protein and Material. arXiv.

[43] Li F., Nian Y., Sun Z., Tao C. (2024). Advancing Biomedicine with Graph Representation Learning: Recent Progress, Challenges, and Future Directions. Brief. Bioinform..

[44] Moor M., Banerjee O., Abad Z.S.H., Krumholz H.M., Leskovec J., Topol E.J., Rajpurkar P. (2023). Foundation Models for Generalist Medical Artificial Intelligence. Nature.

[45] Thirunavukarasu A.J., Ting D.S.J., Elangovan K., Gutierrez L., Tan T.F., Ting D.S.W. (2023). Large Language Models in Medicine. Nat. Med..

[46] Huang S.-C., Pareek A., Seyyedi S., Banerjee I., Lungren M.P. (2020). Fusion of Medical Imaging and Electronic Health Records Using Deep Learning: A Systematic Review and Implementation Guidelines. npj Digit. Med..

[47] Zhou J., Cui G., Hu S., Zhang Z., Yang C., Liu Z., Wang L., Li C., Sun M. (2020). Graph Neural Networks: A Review of Methods and Applications. AI Open.

[48] Scarselli F., Gori M., Tsoi A.C., Hagenbuchner M., Monfardini G. (2009). The Graph Neural Network Model. IEEE Trans. Neural Netw..

[49] Wu Z., Pan S., Chen F., Long G., Zhang C., Yu P.S. (2021). A Comprehensive Survey on Graph Neural Networks. IEEE Trans. Neural Netw. Learn. Syst..

[50] Xiao S., Wang S., Dai Y., Guo W. (2022). Graph Neural Networks in Node Classification: Survey and Evaluation. Mach. Vis. Appl..

[51] Maurya S.K., Liu X., Murata T. (2022). Simplifying Approach to Node Classification in Graph Neural Networks. J. Comput. Sci..

[52] Tsitsulin A., Palowitch J., Perozzi B., Müller E. (2023). Graph Clustering with Graph Neural Networks. J. Mach. Learn. Res..

[53] Wei T., Hou J., Feng R. (2020). Fuzzy Graph Neural Network for Few-Shot Learning. Proceedings of the International Joint Conference on Neural Networks, Glasgow, UK, 19–24 July 2020.

[54] Zhang M., Chen Y. (2018). Link Prediction Based on Graph Neural Networks. arXiv.

[55] Liao R., Li Y., Song Y., Wang S., Hamilton W.L., Duvenaud D.K., Urtasun R., Zemel R.S. (2019). Efficient Graph Generation with Graph Recurrent Attention Networks. arXiv.

[56] Fan X., Gong M., Xie Y., Wang F., Jiang Z., Luo H. (2020). Structured Self-Attention Architecture for Graph-Level Representation Learning. Pattern Recognit..

[57] Wei L., Zhao H., Yao Q., He Z. (2023). Neural Architecture Search for GNN-Based Graph Classification. ACM Trans. Inf. Syst..

[58] Huang R., Li Y., Xie K., Ye H., Li H., Liu Y., Cheng J., Yang S., Li J. Measuring Task Similarity and Its Implication in Fine-Tuning Graph Neural Networks. Proceedings of the AAAI Conference on Artificial Intelligence.

[59] Zhu J., Wang D., Zhang Y., Zhu Y., Chen W., Cheng Y., Liu J. (2023). Geometric Graph Neural Networks on Multi-Omics Data to Predict Cancer Survival Outcomes. Comput. Biol. Med..

[60] Li B., Nabavi S. (2024). A Multimodal Graph Neural Network Framework for Cancer Molecular Subtype Classification. BMC Bioinform..

[61] Li X., Guo R., Zhao S., Qian X. (2023). Causality-Driven Graph Neural Network for Early Diagnosis of Pancreatic Cancer in Non-Contrast Computerized Tomography. IEEE Trans. Med. Imaging.

[62] Zhang Y., Xiong S., Wang Z., Liu Y., Luo H., Li B., Zou Q. (2023). Local Augmented Graph Neural Network for Multi-Omics Cancer Prognosis Prediction and Analysis. Methods.

[63] Chatzianastasis M., Vazirgiannis M., Zhang Z. (2023). Explainable Multilayer Graph Neural Network for Cancer Gene Prediction. Bioinformatics.

[64] Song H., Liu D., Dong S., Zeng L., Wu Z., Zhao P., Zhang L., Liu J. (2023). Identification of Cancer Driver Genes by Integrating Multiomics Data with Graph Neural Networks. Metabolites.

[65] Zhao L., Shen J., Liu L., Yu H., Chen C., Wang Y., Zhang Y., Lin X., Yuan Y. (2023). Biological Knowledge Graph-Guided Investigation of Immune Therapy Response in Cancer with Graph Neural Network. Brief. Bioinform..

[66] Zhang S., Xie W., Li W., Wang L., Feng C. (2023). GAMB-GNN: Graph Neural Networks Learning from Gene Structure Relations and Markov Blanket Ranking for Cancer Classification in Microarray Data. Chemom. Intell. Lab. Syst..

[67] Budak C., Mençik V., Gider V. (2023). Determining Similarities of COVID-19–Lung Cancer Drugs and Affinity Binding Mode Analysis by Graph Neural Network-Based GEFA Method. J. Biomol. Struct. Dyn..

[68] Calderaro S., Lo Bosco G., Vella F., Rizzo R., Rojas I., Valenzuela O., Ruiz, Rojas, Herrera F., Ortuño L.J. (2023). Breast Cancer Histologic Grade Identification by Graph Neural Network Embeddings. Bioinformatics and Biomedical Engineering, Proceedings of the 10th International Work-Conference, IWBBIO 2023, Meloneras, Gran Canaria, Spain, 12–14 July 2023.

[69] Zhu Y., Zhou Y., Liu Y., Wang X., Li J. (2023). SLGNN: Synthetic Lethality Prediction in Human Cancers Based on Factor-Aware Knowledge Graph Neural Network. Bioinformatics.

[70] Yan X., Wang W., Xiao M., Li Y., Gao M. (2024). Survival Prediction Across Diverse Cancer Types Using Neural Networks. arXiv.

[71] Xiao S., Lin H., Wang C., Wang S., Rajapakse J.C. (2023). Graph Neural Networks with Multiple Prior Knowledge for Multi-Omics Data Analysis. IEEE J. Biomed. Health Inform..

[72] Pati S.K., Banerjee A., Manna S. (2023). Gene Selection of Microarray Data Using Heatmap Analysis and Graph Neural Network. Appl. Soft Comput..

[73] Wu W., Liu X., Hamilton R.B., Suriawinata A.A., Hassanpour S. (2023). Graph Convolutional Neural Networks for Histologic Classification of Pancreatic Cancer. Arch. Pathol. Lab. Med..

[74] Peng L., He X., Peng X. (2023). STGNNks: Identifying Cell Types in Spatial Transcriptomics Data Based on Graph Neural Network, Denoising Auto-Encoder, and k-Sums Clustering. Comput. Biol. Med..

[75] Cui Y., Wang Z., Wang X., Zhang Y., Zhang Y., Pan T., Zhang Z., Li S., Guo Y., Akutsu T. (2023). SMG: Self-Supervised Masked Graph Learning for Cancer Gene Identification. Brief. Bioinform..

[76] Wang T., Wang R., Wei L. (2024). AttenSyn: An Attention-Based Deep Graph Neural Network for Anticancer Synergistic Drug Combination Prediction. J. Chem. Inf. Model..

[77] Hernández-Lorenzo L., Paolella R., Mendelson A., Houtman S.J., Kliwinski J., Smedley D., Firth H.V., Campbell J., McRae J.F., Hurles M.E. (2022). On the Limits of Graph Neural Networks for the Early Diagnosis of Alzheimer’s Disease. Sci. Rep..

[78] Ma J., Zhu X., Yang D., Chen J., Wu G. (2020). Attention-Guided Deep Graph Neural Network for Longitudinal Alzheimer’s Disease Analysis. Medical Image Computing and Computer Assisted Intervention—MICCAI 2020, Lima, Peru, 4–8 October 2020.

[79] Kim M., Kim J., Qu J., Huang H., Long Q., Sohn K.-A., Kim D., Shen L. (2021). Interpretable Temporal Graph Neural Network for Prognostic Prediction of Alzheimer’s Disease Using Longitudinal Neuroimaging Data. Proceedings of the 2021 IEEE International Conference on Bioinformatics and Biomedicine, Houston, TX, USA, 9–12 December 2021.

[80] Song X., Mao M., Qian X. (2021). Auto-Metric Graph Neural Network Based on a Meta-Learning Strategy for the Diagnosis of Alzheimer’s Disease. IEEE J. Biomed. Health Inform..

[81] Song T.-A., Chowdhury S.R., Yang F., Jacobs H., El Fakhri G., Li Q., Johnson K.A., Dutta J. (2019). Graph Convolutional Neural Networks for Alzheimer’s Disease Classification. Proceedings of the IEEE International Symposium on Biomedical Imaging, Venice, Italy, 8–11 April 2019.

[82] Zhang Y., He X., Chan Y.H., Teng Q., Rajapakse J.C. (2023). Multi-Modal Graph Neural Network for Early Diagnosis of Alzheimer’s Disease from sMRI and PET Scans. Comput. Biol. Med..

[83] Klepl D., He F., Wu M., Blackburn D.J., Sarrigiannis P.G. (2022). EEG-Based Graph Neural Network Classification of Alzheimer’s Disease: An Empirical Evaluation of Functional Connectivity Methods. IEEE Trans. Neural Syst. Rehabil. Eng..

[84] Sampathkumar V.R. (2021). ADiag: Graph Neural Network Based Diagnosis of Alzheimer’s Disease. arXiv.

[85] Gao J., Liu J., Xu Y., Peng D., Wang Z. (2023). Brain Age Prediction Using the Graph Neural Network Based on Resting-State Functional MRI in Alzheimer’s Disease. Front. Neurosci..

[86] Wang L., Yuan W., Zeng L., Xu J., Mo Y., Zhao X., Peng L. (2022). Dementia Analysis from Functional Connectivity Network with Graph Neural Networks. Inf. Process. Manag..

[87] Cao J., Yang L., Sarrigiannis P.G., Blackburn D.J., Zhao Y. (2024). Dementia Classification Using a Graph Neural Network on Imaging of Effective Brain Connectivity. Comput. Biol. Med..

[88] Hasan M.E., Wagler A. (2024). New CNN and GCN-Based Architecture for AI Applications in Alzheimer’s Disease and Dementia-Stage Classification. AI.

[89] de Haan W., Pijnenburg Y.A.L., Strijers R.L.M., van der Made Y., van der Flier W.M., Scheltens P., Stam C.J. (2009). Functional Neural Network Analysis in Frontotemporal Dementia and Alzheimer’s Disease Using EEG and Graph Theory. BMC Neurosci..

[90] Hu X., Zhang Y., Hadi M., Hu B., Chen Y., Xu J., Liu H., Choi B.Y., Wang F. (2023). Self-Explainable Graph Neural Network for Alzheimer’s Disease and Related Dementias Risk Prediction. arXiv.

[91] Pegolotti L., Pfaller M.R., Rubio N.L., Ding K., Brufau R.B., Darve E., Marsden A.L. (2024). Learning Reduced-Order Models for Cardiovascular Simulations with Graph Neural Networks. Comput. Biol. Med..

[92] de Chou R.S., Sinclair M., Lynch S., Xiao N., Najman L., Vignon-Clementel I., Talbot H. Finite Volume Informed Graph Neural Network for Myocardial Perfusion Simulation. Proceedings of the Medical Imaging with Deep Learning.

[93] Yao T., Pajaziti E., Quail M., Schievano S., Steeden J.A., Muthurangu V. (2024). Image2Flow: A Hybrid Image and Graph Convolutional Neural Network for Rapid Patient-Specific Pulmonary Artery Segmentation and CFD Flow Field Calculation from 3D Cardiac MRI Data. arXiv.

[94] Abisoye O.A. (2024). An Improved Myocardial Infarction Detection Using Convolutional Neural Network and Graph Neural Network Algorithm. Comput. Eng. Appl. J..

[95] Kutluana G., Türker İ. (2024). Classification of Cardiac Disorders Using Weighted Visibility Graph Features from ECG Signals. Biomed. Signal Process. Control.

[96] EPMoghaddam D., Muguli A., Aazhang B. (2023). A Novel Cardiac Arrhythmia Classification Method Using Visibility Graphs and Graph Convolutional Network. Proceedings of the Asilomar Conference on Signals, Systems, and Computers, Pacific Grove, CA, USA, 29 October–1 November 2023.

[97] Rico J.C., Alaeddini A., Faruqui S.H.A., Fisher-Hoch S.P., McCormick J.B. (2024). A Laplacian Regularized Graph Neural Network for Predictive Modeling of Multiple Chronic Conditions. Comput. Methods Programs Biomed..

[98] Zhao C., Xu Z., Baral P., Esposito M., Zhou W. (2024). Multi-Graph Graph Matching for Coronary Artery Semantic Labeling. arXiv.

[99] Lin H., Chen K., Xue Y., Zhong S., Chen L. (2023). Coronary heart disease prediction method fusing domain-adaptive transfer learning with graph convolutional networks (GCN). Sci. Rep..

[100] Zhao C., Xu Z., Hung G.-U., Zhou W. (2023). EAGMN: Coronary Artery Semantic Labeling Using Edge Attention Graph Matching Network. Comput. Biol. Med..

[101] Zhang H., Liu W., Chang S., Wang H., He J., Huang Q. (2024). ST-ReGE: A Novel Spatial-Temporal Residual Graph Convolutional Network for CVD. IEEE J. Biomed. Health Inform..

[102] Walczak L., Noothout J.M.H., Wolterink J.M., Takkenberg J.J.M., van der Bom T., van der Steen A.F.W., de Jaegere P.P.T., Planken R.N., Bouma B.J., van den Boogaard P.J. (2023). 3D Mitral Valve Surface Reconstruction from 3D TEE via Graph Neural Networks. Statistical Atlases and Computational Models of the Heart. Regular and CMRxMotion Challenge Papers.

[103] Duong L.T., Doan T.T.H., Chu C.Q., Nguyen P. (2023). Fusion of Edge Detection and Graph Neural Networks to Classifying Electrocardiogram Signals. Expert Syst. Appl..

[104] Nasrolahzadeh M., Mohammadpoory Z., Haddadnia J. (2024). A Novel Method for Distinction Heart Rate Variability during Meditation Using LSTM Recurrent Neural Networks Based on Visibility Graph. Biomed. Signal Process. Control.

[105] Zhao C., Xu Z., Jiang J., Esposito M., Pienta D., Hung G.-U., Zhou W. (2023). AGMN: Association Graph-Based Graph Matching Network for Coronary Artery Semantic Labeling on Invasive Coronary Angiograms. Pattern Recognit..

[106] Liu M., Gao H., Ji S. (2020). Towards Deeper Graph Neural Networks. Proceedings of the 26th ACM SIGKDD International Conference on Knowledge Discovery & Data Mining, Virtual Event, 23–27 August 2020.

[107] Fan W., Ma Y., Li Q., He Y., Zhao E., Tang J., Yin D. (2019). Graph Neural Networks for Social Recommendation. Proceedings of the World Wide Web Conference, San Francisco, CA, USA, 13–17 May 2019.

[108] Dwivedi V.P., Joshi C.K., Luu A.T., Laurent T., Bengio Y., Bresson X. (2023). Benchmarking Graph Neural Networks. J. Mach. Learn. Res..

[109] You J., Ying Z., Leskovec J. (2020). Design Space for Graph Neural Networks. Adv. Neural Inf. Process. Syst..

[110] Scarselli F., Gori M., Tsoi A.C., Hagenbuchner M., Monfardini G. (2009). Computational Capabilities of Graph Neural Networks. IEEE Trans. Neural Netw..

[111] Li G., Müller M., Ghanem B., Koltun V. Training Graph Neural Networks with 1000 Layers. Proceedings of the 38th International Conference on Machine Learning.

[112] Ying R., Bourgeois D., You J., Zitnik M., Leskovec J. (2019). GNNExplainer: Generating Explanations for Graph Neural Networks. Adv. Neural Inf. Process. Syst..

[113] Wang X., Zhang M. How Powerful Are Spectral Graph Neural Networks. Proceedings of the 39th International Conference on Machine Learning.

[114] Yi L., Su H., Guo X., Guibas L.J. (2017). SyncSpecCNN: Synchronized Spectral CNN for 3D Shape Segmentation. Proceedings of the IEEE Conference on Computer Vision and Pattern Recognition, Honolulu, HI, USA, 21–26 July 2017.

[115] Defferrard M., Bresson X., Vandergheynst P. Convolutional Neural Networks on Graphs with Fast Localized Spectral Filtering. Proceedings of the 30th International Conference on Neural Information Processing Systems.

[116] Yao L., Mao C., Luo Y. Graph Convolutional Networks for Text Classification. Proceedings of the AAAI Conference on Artificial Intelligence.

[117] Liu J., Ong G.P., Chen X. (2022). GraphSAGE-Based Traffic Speed Forecasting for Segment Network with Sparse Data. IEEE Trans. Intell. Transp. Syst..

[118] Veličković P., Cucurull G., Casanova A., Romero A., Liò P., Bengio Y. Graph Attention Networks. Proceedings of the International Conference on Learning Representations.

[119] Gilmer J., Schoenholz S.S., Riley P.F., Vinyals O., Dahl G.E., Schütt K.T., Chmiela S., von Lilienfeld O.A., Tkatchenko A., Tsuda K., Müller K.-R. (2020). Message Passing Neural Networks. Machine Learning Meets Quantum Physics.

[120] Cai S., Li Y., Zhang S., Chen Z., Pu S. (2021). Rethinking Graph Neural Architecture Search from Message-Passing. Proceedings of the IEEE/CVF Conference on Computer Vision and Pattern Recognition, Virtual Event, 19–25 June 2021.

[121] Tang M., Li B., Chen H. (2023). Application of Message Passing Neural Networks for Molecular Property Prediction. Curr. Opin. Struct. Biol..

[122] Wang X., Ji H., Shi C., Wang B., Ye Y., Cui P., Yu P.S. (2019). Heterogeneous Graph Attention Network. Proceedings of the World Wide Web Conference, San Francisco, CA, USA, 13–17 May 2019.

[123] Lo W.W.Y., Yang X., Wang Y. (2022). E-GraphSAGE: A Graph Neural Network Based Intrusion Detection System for IoT. Proceedings of the NOMS 2022—2022 IEEE/IFIP Network Operations and Management Symposium, Budapest, Hungary, 25–29 April 2022.

[124] Wang S., Wu Z., Liu Y., Yao L., Zhang H., Wu J. (2023). An Improved Graph Isomorphism Network for Accurate Prediction of Drug–Drug Interactions. Mathematics.

[125] Lu Z., Lv W., Xie Z., Du B., Xiong G., Sun L., Wang H. (2022). Graph Sequence Neural Network with an Attention Mechanism for Traffic Speed Prediction. ACM Trans. Intell. Syst. Technol..

[126] Zhang Y., Wang X., Shi C., Jiang X., Ye Y. (2022). Hyperbolic Graph Attention Network. IEEE Trans. Big Data.

[127] Cordonnier J.-B., Loukas A., Jaggi M. (2020). Multi-Head Attention: Collaborate Instead of Concatenate. arXiv.

[128] Gong L., Cheng Q. (2019). Exploiting Edge Features for Graph Neural Networks. Proceedings of the IEEE/CVF Conference on Computer Vision and Pattern Recognition, Long Beach, CA, USA, 15–20 June 2019.

[129] Yin C., Wu K., Che Z., Jiang B., Xu Z., Tang J. (2021). Hierarchical Graph Attention Network for Few-Shot Visual-Semantic Learning. Proceedings of the IEEE/CVF International Conference on Computer Vision, Montreal, QC, Canada, 10–17 October 2021.

[130] Ghose A., Zhang V., Zhang Y., Li D., Liu W., Coates M. (2021). Generalizable Cross-Graph Embedding for GNN-Based Congestion Prediction. Proceedings of the IEEE/ACM International Conference on Computer-Aided Design, Munich, Germany, 1–4 November 2021.

[131] Kim B.-H., Ye J.C., Kim J.-J. (2021). Learning Dynamic Graph Representation of Brain Connectome with Spatio-Temporal Attention. Adv. Neural Inf. Process. Syst..

[132] Yun S., Jeong M., Kim R., Kang J., Kim H.J. (2019). Graph Transformer Networks. Adv. Neural Inf. Process. Syst..

[133] Dwivedi V.P., Bresson X. A Generalization of Transformer Networks to Graphs. Proceedings of the AAAI Workshop on Deep Learning on Graphs: Methods and Applications.

[134] Rampášek L., Galkin M., Dwivedi V.P., Luu A.T., Wolf G., Beaini D. (2022). Recipe for a General, Powerful, Scalable Graph Transformer. Adv. Neural Inf. Process. Syst..

[135] Chen Y., Zhang F., Wang M., Zekelman L.R., Cetin-Karayumak S., Xue T., Zhang C., Song Y., Rushmore J., Makris N. (2025). TractGraphFormer: Anatomically Informed Hybrid Graph CNN-Transformer Network for Interpretable Sex and Age Prediction from Diffusion MRI Tractography. Med. Image Anal..

[136] Chechkin A., Pleshakova E., Gataullin S. (2025). A Hybrid Neural Network Transformer for Detecting and Classifying Destructive Content in Digital Space. Algorithms.

[137] Xie Y., Xu Z., Zhang J., Wang Z., Ji S. (2023). Self-Supervised Learning of Graph Neural Networks: A Unified Review. IEEE Trans. Pattern Anal. Mach. Intell..

[138] Kipf T.N., Welling M. Semi-Supervised Classification with Graph Convolutional Networks. Proceedings of the International Conference on Learning Representations.

[139] Yang L., Cao X., He D., Wang C., Wang X., Zhang W. (2020). Toward Unsupervised Graph Neural Network: Interactive Clustering and Embedding via Optimal Transport. Proceedings of the IEEE International Conference on Data Mining, Sorrento, Italy, 17–20 November 2020.

[140] Ma H., Rong Y., Huang J., Wu L., Cui P., Pei J., Zhao L. (2022). Graph Neural Networks: Scalability. Graph Neural Networks: Foundations, Frontiers, and Applications.

[141] Zeng H., Zhou H., Srivastava A., Kannan R., Prasanna V. (2021). Accurate, Efficient and Scalable Training of Graph Neural Networks. J. Parallel Distrib. Comput..

[142] Liu X., Yan M., Deng L., Li G., Ye X., Fan D. (2022). Sampling Methods for Efficient Training of Graph Convolutional Networks: A Survey. IEEE/CAA J. Autom. Sin..

[143] Chen J., Ma T., Xiao C. FastGCN: Fast Learning with Graph Convolutional Networks via Importance Sampling. Proceedings of the International Conference on Learning Representations.

[144] Zhang C., Song D., Huang C., Swami A., Chawla N.V. (2019). Heterogeneous Graph Neural Network. Proceedings of the 25th ACM SIGKDD International Conference on Knowledge Discovery & Data Mining, Anchorage, AK, USA, 4–8 August 2019.

[145] Poli M., Massaroli S., Park J., Yamashita A., Asama H., Park J. (2019). Graph Neural Ordinary Differential Equations. arXiv.

[146] Ramadan T., Lahiry A., Islam T.Z. (2023). Novel Representation Learning Technique Using Graphs for Performance Analytics. Proceedings of the IEEE International Conference on Machine Learning and Applications, Jacksonville, FL, USA, 15–17 December 2023.

[147] Singh Y., Farrelly C.M., Hathaway Q.A., Leiner T., Jagtap J., Carlsson G.E., Erickson B.J. (2023). Topological data analysis in medical imaging: Current state of the art. Insights Into Imaging.

[148] Tan C.W., Yu P.-D., Chen S., Poor H.V. (2025). DeepTrace: Learning to Optimize Contact Tracing in Epidemic Networks with Graph Neural Networks. IEEE Trans. Signal Inf. Process. Netw..

[149] Hang C.N., Yu P.-D., Chen S., Tan C.W., Chen G. (2023). MEGA: Machine Learning-Enhanced Graph Analytics for Infodemic Risk Management. IEEE J. Biomed. Health Inform..

[150] Smirnov D. (2022). Deep Learning on Geometry Representations. Ph.D. Thesis.

[151] Cai C., Vlassis N., Magee L., Ma R., Xiong Z., Bahmani B., Wong T.-F., Wang Y., Sun W. (2023). Equivariant Geometric Learning for Digital Rock Physics: Estimating Formation Factor and Effective Permeability Tensors from Morse Graph. Int. J. Multiscale Comput. Eng..

[152] Tandon K., Gould J., Bhatia T., Dominici F., Ribeiro A., Battiloro C. (2026). Consistent Geometric Deep Learning via Hilbert Bundles and Cellular Sheaves. arXiv.

