# Supplementary material for: Graph Neural Networks for Medical Imaging Analysis and Biological Data: Integrating Topology, Geometry, Radiomics, and Generative AI
Source: Bioengineering (Basel). 2026 May 29;13(6):638. doi: 10.3390/bioengineering13060638 (PMC13295888; doi:10.3390/bioengineering13060638)
Supplement: Supplementary file 1 [file bioengineering-13-00638-s001.zip › bioengineering-4310208-supplementary.pdf]

bioengineering

MDPI

Table S1. Taxonomy of GNN model families and graph-learning strategies relevant to biomedical imaging analysis.

Taxonomy Domain	Model, Strategy, or Task	Core Technical Mechanism	Biomedical Imaging Relevance	References
**Spectral graph convolution**	Spectral CNN	Extends convolution to graph-structured data using graph Fourier transforms and spectral filtering	Useful when imaging-derived graphs have meaningful frequency structure, such as brain networks, anatomical adjacency graphs, or radiomics graphs	[113,114]
	ChebNet	Uses Chebyshev polynomial approximations to avoid explicit eigen-decomposition of the graph Laplacian	Enables more efficient spectral filtering for larger biomedical graphs	[115]
	Graph convolutional network	Uses localized first-order spectral approximations for efficient graph convolution	Applicable to region-adjacency graphs, molecular graphs, patient-similarity graphs, and imaging-derived structured data	[116]
**Spatial graph convolution**	GraphSAGE	Samples and aggregates local neighborhoods to generate inductive node embeddings	Useful for large biomedical graphs and settings where new patients, cells, or regions may appear at inference	[117,123]
	Graph attention network	Learns attention weights over neighboring nodes during message passing	Useful when different anatomical regions, cells, biomarkers, or graph neighbors contribute unequally to prediction	[118,122,126]
	Message passing neural network	General framework in which node and edge information are propagated and updated across graph neighborhoods	Useful for molecular graphs, biological networks, pathology cell graphs, and region-level imaging graphs	[119–121]
**Aggregation strategy**	Graph isomorphism network	Uses highly discriminative aggregation to distinguish graph structures	Relevant for graph classification tasks where subtle structural differences may encode disease phenotype	[124]
**Attention mechanism**	Multi-head attention	Learns multiple attention patterns in parallel to capture complementary graph relationships	May improve modeling of heterogeneous or multimodal biomedical graphs	[127]
	Edge attention	Incorporates edge-specific information into attention or message passing	Useful when edge attributes encode distance, connectivity strength, anatomical adjacency, or molecular interaction type	[128]
	Hierarchical attention	Applies attention at multiple graph levels, such as node, subgraph, or graph level	Relevant for multiscale imaging phenotypes, including cellular, tissue, organ, and patient-level graphs	[129]

	Cross-graph attention	Learns correspondences or dependencies across multiple graphs	Useful for graph matching, longitudinal comparison, multimodal alignment, or patient-to-patient similarity analysis	[130]
	Temporal attention	Weights graph states across time	Relevant for longitudinal imaging, dynamic brain connectivity, ECG analysis, and disease-progression modeling	[131]
**Graph-structure task**	Node classification/clustering	Predicts labels for nodes or identifies groups of similar nodes	May classify cells, anatomical regions, lesions, or disease-associated subregions	[53–55]
	Link prediction/edge classification	Predicts missing or clinically meaningful relationships between nodes	Useful for biomarker interaction prediction, molecular networks, functional connectivity, or missing-edge imputation	[42–44]
	Graph classification/similarity analysis	Predicts labels or similarities for entire graphs	Relevant for patient-level diagnosis, prognosis, disease staging, or imaging phenotype comparison	[56–58]
**Hybrid architecture**	Hybrid Graph–Transformer models	Combines local graph message passing with global transformer-based self-attention, often using structural or positional encodings	Promising for multiscale imaging tasks requiring both local anatomical adjacency and long-range biological dependencies	[127,130–136]

Table S2. Training and scalability considerations for biomedical GNNs.

Topic	Method	Biomedical Relevance	Key Limitation	References
**Supervised learning**	Uses labeled node-, edge-, or graph-level outcomes	Diagnosis, prognosis, segmentation, treatment response	Requires large, reliable labels	[137]
**Semi-supervised learning**	Combines limited labels with graph structure and unlabeled data	Useful when only subsets of images, regions, or patients are labeled	Sensitive to label noise and graph construction	[138]
**Unsupervised/self-supervised learning**	Learns embeddings using reconstruction, clustering, contrastive, or autoencoding objectives	Pretraining and representation learning from unlabeled biomedical graphs	Embeddings require clinical validation	[139]
**Sampling-based training**	Samples neighborhoods or subgraphs to reduce computation	Enables large pathology, molecular, patient, or imaging-derived graphs	May miss long-range or rare disease-relevant structure	[140–142]
**Layer-wise propagation**	Samples nodes layer-by-layer to reduce neighborhood explosion	Improves scalability of deeper/larger GNNs	Approximation depends on sampling distribution	[140,141,143]
**Temporal GNNs**	Models dynamic nodes, edges, or graph states	Longitudinal imaging, ECG, disease progression, dynamic connectivity	Requires time-resolved data and missingness handling	[142]
**Heterogeneous GNNs**	Models multiple node and edge types	Multimodal imaging-clinical-genomic graphs	Requires careful graph schema design	[144]
**Graph Neural ODEs**	Models continuous graph feature evolution	Continuous-time biomedical and physiologic dynamics	More complex optimization and interpretation	[145]

https://doi.org/10.3390/bioengineering13060638

Table S3. Mathematical operators and representation-learning roles in topology- and geometry-aware GNNs.

Ref	Mathematical Concept	Formal Object	Representation-Learning Role	Biomedical Imaging Relevance	Practical Limitation
[113–116]	Spectral graph representation	Graph Laplacian $L = D - A$; normalized Laplacian $L_{\text{norm}} = I - D^{-1/2}AD^{-1/2}$; eigendecomposition $L = U\Lambda U^{\top}$	Defines graph Fourier basis and learnable graph-frequency filters	Brain connectivity, vascular graphs, region-adjacency graphs, radiomics graphs	Eigendecomposition can be computationally expensive; spectral filters may transfer poorly across variable patient-specific graphs
[7–17]	Persistent homology	Filtration $X_0 \subseteq X_1 \subseteq \cdots \subseteq X_t$; homology groups H_0, H_1, H_2; birth–death intervals	Encodes multi-scale connected components, loops, and voids	Tumor heterogeneity, necrosis, glandular/tissue architecture, cortical folding, vascular branching	Can be computationally costly for dense volumetric data; requires vectorization or differentiable approximation
[18–22]	Hodge-Laplacian learning	Boundary operators B_k; $L_k = B_k^{\top}B_k + B_{k+1}B_{k+1}^{\top}$	Extends message passing and signal processing to edges, faces, and higher-order simplices	Tissue compartments, cell neighborhoods, vascular surfaces, cortical surfaces, multi-region interactions	Requires construction of simplicial/cellular complexes; may increase memory and preprocessing burden
[25–33]	Equivariant GNNs	Group equivariance $f(g \cdot x) = g \cdot f(x)$; permutation equivariance $f(PAP^{\top}, PX) = Pf(A, X)$	Preserves known symmetries during representation learning	Pathology rotation, anatomical orientation variability, molecular geometry, arbitrary node ordering	Requires correct choice of symmetry group; overly restrictive equivariance assumptions may limit flexibility
[23,24,34,35]	Curvature/geodesic geometry	Ricci or Ollivier–Ricci curvature; shortest-path or manifold distances	Weight aggregation according to local geometry or graph manifold structure	Branching vessels, airway trees, cortical surfaces, anatomical meshes	Curvature and geodesic estimates can be noisy or computationally expensive

Table S4. Representative open-source software resources for GNN-based biomedical imaging and graph-learning workflows.

Resource/Repository	Primary Use	Relevance to Manuscript	Link
**PyTorch Geometric**	General GNN implementation library in PyTorch	Provides widely used tools for building and training GNNs on structured data, including many message-passing architectures	https://github.com/pyg-team/pytorch_geometric
**Deep Graph Library**	Scalable deep learning library for graphs	Supports high-performance and scalable graph deep learning across common deep learning frameworks	https://github.com/dmlc/dgl
**MONAI**	Medical imaging AI framework	Supports deep learning workflows for healthcare imaging preprocessing, training, evaluation, and deployment	https://github.com/project-monai/monai
**PyRadiomics**	Radiomics feature extraction	Provides standardized extraction of radiomic features from medical images	https://github.com/AIM-Harvard/pyradiomics

https://doi.org/10.3390/bioengineering13060638

		that can be used as node-, edge-, or graph-level features	
**Equivariant Graph Neural Networks repository**	Curated collection of equivariant GNN implementations	Relevant to geometry-aware and equivariant GNNs that preserve symmetry properties in graph learning	https://github.com/kthrn22/Equivariant-Graph-Neural-Networks
**Curvature Graph Neural Network**	Curvature-aware GNN implementation	Relevant to curvature-based GNNs and geometric graph aggregation	https://github.com/GeoX-Lab/CGNN
**Neural Sheaf Diffusion**	Sheaf neural network implementation	Relevant to sheaf neural networks and diffusion over graph/sheaf structures	https://github.com/twitter-research/neural-sheaf-diffusion
**TOGL**	Topological graph layer implementation	Relevant to topology-aware GNNs that incorporate persistent-homology-based graph representations	https://github.com/BorgwardtLab/TOGL
**DIG: Dive into Graphs**	Graph deep learning research library	Provides graph learning benchmarks, models, and research tools useful for prototyping GNN methods	https://github.com/divelab/DIG

These repositories are provided as representative implementation resources for readers interested in applying GNN-related methods. They are not exhaustive and should not be interpreted as clinical-grade software. Users should verify installation requirements, licensing, maintenance status, and compatibility with their datasets and regulatory environment before use.

https://doi.org/10.3390/bioengineering13060638
